# Comparative Toll-like receptor profiling reveals breed-specific innate immune architecture following Newcastle disease vaccination

**DOI:** 10.1016/j.psj.2026.107908

**Published:** 2026-10-05

**Authors:** Patel Nikunjkumar, Gautham Kolluri, Abinaya Kaliappan, Shiva Kumar Kurva, Aditi Vats, Saravanan Ramakrishnan, Jagbir Singh Tyagi

**Affiliations:** a Immunology Section, ICAR-Indian Veterinary Research Institute, Bareilly, UP, 243122, India; b Molecular Physiology Laboratory, Division of Avian Physiology and Reproduction, ICAR-Central Avian Research Institute, Bareilly, UP, 243122, India

**Keywords:** Chicken, Newcastle disease virus, Innate immunity, Toll-like receptors, Expression analysis

## Abstract

The present study investigated the expression profiles of six TLR genes (TLR2A, TLR3, TLR4, TLR5, TLR7 and TLR21) in peripheral blood mononuclear cells (PBMCs) following genotype II ND vaccination across fifteen genetically diverse chicken populations representing exotic, indigenous, fancy, synthetic and broiler parent lines. Relative gene expression was quantified by qPCR, and receptor expression data were integrated into five novel composite immune indices, namely the Viral Recognition Index (VRI), Bacterial Recognition Index (BRI), Immune Diversity Score (IDS), Shannon TLR Diversity Index (STDI), and Immune Breadth Index (IBI). Principal component analysis (PCA), hierarchical clustering and correlation analyses were further employed to characterize breed-specific innate immune architecture. Significant breed-dependent variation (P < 0.001) was observed for all six TLRs, demonstrating substantial genetic diversity in early pathogen recognition. Indigenous breeds, particularly Aseel Red and Kadaknath, exhibited broader activation of bacterial and viral recognition receptors, whereas White Frizzle showed the highest TLR2A expression and Frizzle displayed comparatively greater TLR5 and TLR7 expression among the fancy breeds. Synthetic populations exhibited intermediate but line-specific immune activation, while the broiler parent line (BCSFL) consistently demonstrated comparatively lower expression across most TLRs. PCA demonstrated pronounced breed-specific clustering of innate immune responses, with PC1 alone explaining 55.9% of the total variance, suggesting that coordinated TLR expression constituted the major source of immunological variation among chicken breeds. While correlation and hierarchical clustering analyses identified coordinated activation of bacterial (TLR2A, TLR4 and TLR5) and viral (TLR3, TLR7 and TLR21) sensing pathways in indigenous populations, whereas the Shannon TLR Diversity Index remained relatively conserved across most breeds. ND vaccine-induced TLR mediated immunity is characterized by functional specialization rather than hierarchical superiority, with distinct chicken genetic groups exhibiting differential investment in pathogen recognition pathways. The composite immune indices proposed herein provide a quantitative systems-level framework for evaluating innate immune architecture and represent promising hypothesis-generating biomarkers for comparative immunogenetics, precision breeding, and vaccine evaluation in poultry, warranting further validation through independent populations and virulent NDV challenge studies.

## Introduction

Infectious diseases affecting poultry are continuously hindering the growth of poultry industry. Continuous evolution of pathogens and antibiotic resistant strains create challenges in the control of infectious diseases. Newcastle disease (ND) is distributed worldwide and affects a wide range of avian species, causing high mortality and severe clinical symptoms (Alexander, 2001). ND virus strains which is capable of infecting more than 200 avian species, remains a major threat to the poultry industry and has been associated with mortality rates of up to 40.22%, resulting in substantial economic losses (Kolluri et al., 2024). Despite widespread vaccination, Newcastle disease (ND) remains a major constraint to poultry production worldwide, with marked variation in vaccine responsiveness and disease susceptibility among chicken breeds. The mechanisms underlying this heterogeneity are not fully understood, limiting the development of breed-informed vaccination and breeding strategies.

The genetic makeup of the bird has influence on production capacity, health and disease resistance. Susceptibility and resistance to various pathogens varies between chicken breeds and strains (Broom and Kogut, 2019). Host resistance to infectious diseases is not solely determined by the magnitude of immune activation but also by the strategic allocation of immune resources. Mounting and maintaining immune responses impose substantial energetic and physiological costs, leading to evolutionary trade-offs between pathogen resistance, growth, reproduction, and metabolic performance (Lochmiller and Deerenberg, 2000; Schmid-Hempel, 2003). Consequently, natural selection and artificial breeding have shaped distinct immune strategies across chicken populations, resulting in genetic variation in immune responsiveness rather than a universally superior immune phenotype (Kaiser, 2010). Indigenous chicken breeds, having evolved under diverse pathogen-rich environments, are hypothesized to maintain greater innate immune preparedness, whereas highly selected commercial lines have been intensively bred for production traits, potentially altering immune investment and disease resilience (Cheng and Muir, 2005; Lamont et al., 2002 ). These immune trade-offs are likely reflected in the diversity and balance of Toll-like receptor (TLR)-mediated pathogen recognition pathways, which determine the efficiency of early pathogen sensing, downstream inflammatory signalling, and vaccine-induced immunity (Akira et al., 2006; Kawai and Akira, 2010).

The immune system has two major arms namely innate and adaptive immunity. The innate immune response is the first line of defense against invading pathogens and is required for the triggering of efficient adaptive immune response thereby plays primary role in disease resistance. The Toll-like receptor (TLR) signaling pathway is a key and well-characterized component of the innate immune system, in which TLRs, acting as pattern recognition receptors (PRRs), provide an evolutionarily conserved first line of defense by recognizing pathogen-associated molecular patterns (PAMPs) and initiating immune responses against invading pathogens (Kawai et al., 2024) and and transportation stress (Naldurtiker et al., 2023). In chicken, ten TLR types have been reported of which TLR1, TLR2, TLR4, TLR5 are resident of cellular surface and TLR3, TLR7, TLR15, TLR21 are intracellular (Kannaki et al., 2011; Neerukonda and Katneni, 2020). These receptors initiate a series of cellular downstream signaling aimed to induce production of pro-inflammatory, anti-inflammatory, Th1-Th2 cytokines, type-I interferons (IFNs), which prompt innate immunity and further guide the development of adaptive immunity (Nawab et al., 2019). TLR1A, TLR1B, TLR2A, TLR2B, TLR3, TLR4, TLR5, TLR7, TLR15, and TLR21 have been reported in chicken (Gupta et al., 2015). Toll-like receptors expression pattern on immune cells is an important factor to be considered when determining the immunological status of the host (Kogut et al., 2006). Recently, Świderská et al. (2018) reported that chicken TLRs exhibit nine-time higher nucleotide diversity as compared to human TLRs, which might be a contributing factor for disease resistance/susceptibility. Higher TLR gene polymorphisms in chicken could be exploited to improve the health through breeding. Indigenous chicken breeds are gaining attention for their higher tolerance to certain diseases despite their frequent exposure to marshy areas where the incidence of potential pathogens is relatively high; this might be due to the elicitation of strong innate immune responses (Kolluri et al., 2014). Indian native chicken breeds are considered to have better immune competence and disease tolerance (Prabhu et al., 2020; Yadav et al., 2018). Previously, some research groups reported the comparative expression of PRRs including TLRs in various breeds of chicken (Haunshi et al., 2017; Ramasamy et al., 2010, 2011) and duck (Kolluri et al., 2014). Recent unpublished findings from our lab indicated higher expression levels of TLR7 and IFN-γ in native Aseel breed contributed for higher disease resilience to velogenic Newcastle Disease virus.

Innate immune recognition through Toll-like receptors (TLRs) is pivotal in shaping the magnitude and quality of subsequent adaptive responses to vaccination. Because chicken breeds differ substantially in their genetic background and evolutionary history, they are likely to possess distinct innate immune architectures that influence their capacity to sense and respond to vaccine antigens. Nevertheless, comparative information on the early TLR-mediated responses to ND vaccination across diverse chicken genetic resources remains scarce. Therefore it is essential for identifying immune traits that can be incorporated into breeding programmes aimed at improving disease resistance without compromising productivity (Berghof et al., 2019; Jie and Liu, 2011; Looi et al., 2018). Knowledge on the expression profile of TLRs would contribute to better understanding of host pathogen interactions in indigenous and commercial chicken. We therefore hypothesized that indigenous, exotic, fancy, and crossbred/synthetic chicken lines exhibit breed-specific patterns of TLR expression following ND vaccination, reflecting inherent differences in innate immune preparedness and responsiveness. To test this hypothesis, the present study characterized the early transcriptional responses of key TLRs in peripheral blood mononuclear cells which are widely recognized as representative systemic immune cells following ND vaccination acros a diverse panel of chicken breeds. To the best of our knowledge, this is the first study to report comparative TLR expression profiling in the PBMCs from different fancy, cross, indigenous and exotic chicken breeds.

## Materials and methods

### Experimental animals

Various chicken breeds and varieties representing fancy, broiler, layer, dual-purpose, indigenous, and exotic genetic groups (Table 1) were selected for the study. The birds were maintained at the research farms of ICAR–Central Avian Research Institute, Izatnagar, Uttar Pradesh, India, where these genetic resources have been maintained, bred, and utilized for research and genetic improvement over several decades. Birds were reared under standard institutional management practices in an open-house battery cage system, with routine access to feed and sterilized drinking water and management appropriate for their respective production types. Birds aged 35–40 weeks, corresponding to the peak egg-production period, were used for the experiment.

**Table 1:** List of chicken breeds and their codes used in the experiment.

Breed/Variety	Breed/Variety type	Breed/Variety/Line code Assigned
**Unvaccinated/Control**		
White Leghorn	Exotic	WLHC
**Vaccinated**		
White Leghorn	Exotic	WLH
Rhode Island Red	Exotic	RIR
Aseel Peela (Red plumage variety)	Indigenous	AR
Aseel Kagar (Black plumage variety)	Indigenous	AB
Nicobari	Indigenous	NCB
Kadaknath	Indigenous	KN
Frizzle	Fancy	FRZ
Silkie	Fancy	SLK
White Frizzle	Fancy	WFRZ
CARIRED	Cross	CR
CARISHYAMA	Cross	CSH
CARIBRODHANARAJA Broiler	Cross	CDB
Synthetic Female Line of Broiler	Cross (broiler parent lines)	SFLB
Coloured Synthetic Female Line of Broiler	Cross (broiler parent lines)	BCSFL
Coloured Synthetic Male Line of Broiler	Cross (broiler parent lines)	BCSML

Because the study included 15 different chicken breeds, whereas specific-pathogen-free (SPF) birds were available for only one breed, the experimental birds could not be maintained entirely under SPF conditions. Accordingly, all birds were reared under conventional research-farm conditions. Clinically healthy birds were selected based on routine veterinary observation, with no apparent signs of disease at the time of experimentation. However, only clinically healthy birds were selected, and all breeds were maintained under the same institutional management conditions. The possibility that differences in background microbial exposure or subclinical immune stimulation contributed to variation in basal TLR expression cannot therefore be completely ruled out and is acknowledged as a potential confounding factor. The birds had received the institute’s routine vaccination programme from hatch onwards, including vaccination against Marek’s disease, Newcastle disease (ND), infectious bursal disease (IBD), and fowl pox, with booster doses administered according to the institutional schedule. The vaccination programme comprised HVT FC-126 for Marek’s disease; LaSota (lentogenic), R2B (mesogenic), and killed LaSota vaccines for ND; intermediate-plus and killed vaccines for IBD; and live fowl pox vaccine.

### Animal ethics statement

All procedures involving the handling and care of animals during management and sample collection were approved by the Institutional Animal Ethics Committee, Izatnagar, India (Approval No. 452/01/ab/CPCSEA), and were conducted in accordance with the guidelines of the Committee for the Purpose of Control and Supervision of Experiments on Animals (CPCSEA), a statutory body established under Chapter 4, Section 15(1) of the Prevention of Cruelty to Animals Act, 1960, India. This study did not involve human participants and complies with the ARRIVE 3.0 guidelines for reporting animal research.

### Virus administration and sample collection

For the experimental purpose, clinically healthy birds of 6 for each breed were selected and vaccine virus was administered with LaSota lentogenic strain of Genotype-II having ≥10^6^ EID_50_ (Embryonic Infective Dose) per each dose through intra-ocular route. For the control group, six chickens belonging to White Leghorn breed alone were administered normal saline through the same route and served as the unvaccinated control. Blood samples were collected on 48 h post-vaccination as a predefined early post-vaccination sampling point. One bird per chicken breed was considered as biological replicate and 6 birds (6 biological replicates) from each breed were utilized. Around 3 mL of blood was collected aseptically from jugular vein into a syringe containing 20 IU/mL heparin as an anti-coagulant.

### Isolation of peripheral blood mononuclear cells

The isolation of PBMCs was done as described previously (Bashir et al., 2019). Briefly, blood was transferred to a 15 mL sterile centrifuge tube containing equal volume of Ficoll-Histopaque 1.077 (Sigma-Aldrich, USA) and centrifuged at 500 × g for 45 min. The interface containing the PBMCs was transferred to a new centrifuge tube and washed twice with phosphate-buffered saline (PBS, pH 7.2) and resuspended in 1 mL of PBS. Cell viability was determined by trypan blue dye exclusion method, and approximately 2 × 10^6^ cells from each samples were stored in 1 mL of TRIzol (Takara, Japan) and kept at −80 °C until RNA extraction.

### Total RNA extraction and cDNA synthesis

The total RNA was extracted from the PBMCs using TRIzol-Reagent (Takara, Japan) as per manufacturer’s guidelines. Briefly, 250 µL of chloroform was added in TRIzol containing cells mixture and centrifuged at 12,000 × g for 20 min at 4 °C for phase separation. The aqueous phase containing RNA was precipitated with 500 µL of isopropanol by gentle mixing followed by centrifugation at 12,000 × g for 10 min at 4 °C. The supernatant was removed, and the RNA pellet was washed with one mL of 75% ethanol by centrifuging at 7,500 × g for 5 min at 4 °C. Ethanol was carefully removed and the RNA pellet was dissolved in 20 µL of RNase-free water and stored at −80°C until further use. RNA concentration and purity were determined by measuring the absorbance at 260 nm and 280 nm and calculating the A_260_/A_280_ ratio using microvolume UV-VIS spectrophotometer (NanoDrop, Thermo Scientific, USA). The purity and concentration of total RNA was further validated by flourometric detection using QUBIT 3.0 Flourometer (Invitrogen, USA). First strand cDNA was synthesized from 2 µg of RNA using a RevertAid First Strand cDNA Synthesis Kit (Thermo Scientific, USA) and random hexamers (Thermo Scientific, USA) following manufacturer’s instructions.

### Determination of expression level and stability of housekeeping genes

The stability of the suitable housekeeping gene was optimized before proceeding with the actual experimental samples analysis. The expression levels of reference genes (Glyceraldehyde-3-phosphate dehydrogenase (GAPDH) and β-actin were measured by calculating the Ct value of PBMCs derived from all the experimental birds by real-time PCR. RefFinder online software was used to rank the stability of reference gene by employing different algorithms viz., GeNorm, Bestkeeper© and delta CT (Pfaffl et al., 2004; Silver et al., 2006; Vandesompele et al., 2002) to select the suitable house keeping gene.

### Quantitative analysis of Toll-like receptors using real-time PCR

All the gene specific primers used in this study were custom synthesized from Integrated DNA Technologies, India. The mRNA expression levels of TLR2A, TLR3, TLR4, TLR5, TLR7 and TLR21 were determined by quantitative real-time PCR as per minimum information for quantitative experiments (MIQE) guidelines using QuantiFast SYBR Green PCR Kit (Qiagen, USA). Amplification and detection of target genes were performed on AriaMx Real time PCR system (Agilent, USA). A total of 20 µL reaction mixture consisted of 2 μL of 1:10 diluted cDNA in nuclease free water, 10 μL QuantiFast SYBR Green Master Mix, 0.5 μL of each forward and reverse primers (10 pmol), and nuclease-free water up to a volume of 20 μL and was prepared in a flat cap optically clear reaction vessel. Thermal conditions involved a pre-incubation at 95°C for 5 min followed by 40 cycles each of 94 °C for 30 sec, 60 °C for 45 sec, and 70 °C for 45 sec. The melt curve analysis was performed with a single cycle of 95 °C for 30 sec, 55 °C for 5 sec, and 95°C for 30 sec to check the specificity of amplicon. The gene specific primer sequences, amplicon size and accession numbers are listed in Table 2.

**Table 2:** Oligonucleotide sequences and their annealing temperatures used in the study.

Primer	Sequence (5′−3′)	Amplicon size (bp)	Annealing temp. (°C)	References
GAPDH	F :agcacccgcatcaaagg	283	60	(Yang et al., 2013)
	R :catcatcccagcgtcca			
β-actin	F : gcaccacactttctacaatag	184	60	(Kannaki et al., 2015)
	R : acgaccagaggcatacagg			
TLR2A	F :atcctgctggagcccattcagag	108	60	(St. Paul et al., 2013)
	R :ttgctcttcatcaggaggccactc			
TLR3	F :tcagtacatttgtaacaccccgcc	256	60	(St. Paul et al., 2013)
	R :ggcgtcataatcaaacactcc			
TLR4	F :gctgggcaaagtgaaaagag	225	60	(Quan et al., 2017)
	R :taagaacagcccgttcatcc			
TLR5	F :ttcttgcaacctcacaggtgttcc	208	60	(St. Paul et al., 2013)
	R :caggtccaagacacgaagatt			
TLR7	F :agagactggcttccaggaca	219	60	(Nang et al., 2011)
	R :cagctgaacataccgggact			
TLR21	F :cctgcgcaagtgtccgctca	167	60	(St. Paul et al., 2013)
	R :gccccaggtccaggaagcag			

A total of two technical replicates were maintained for each biological replicate (one single chicken). Relative quantification of gene expression was performed using the comparative Ct method, with Ct values. The relative threshold cycle (Ct-the PCR cycle at which amplification becomes significant enough to cross the predetermined threshold) values were normalized to the endogenous control. The Ct values of the PCRs that were in duplicate were averaged. The ∆Ct represents the Ct value of the house keeping gene subtracted from the Ct value of the target gene (Livak and Schmittgen, 2001). The relative log fold change (fc) was calculated with the WLH breed as a control and the relative fold change in all other breeds was studied in terms of up-regulation or down regulation. In addition, TLRs are expressed as 40–ΔCt mean values as described previously (Kolluri et al., 2014; MacKinnon et al., 2009; Ramasamy et al., 2010). The higher 40–ΔCt value can be interpreted as a numerical value indicating greater gene expression. This provides a comprehensive overall expression on individual breed basis.

### Exploartory TLR repertoire-based innate immune indices

To summarize the expression of viral-sensing and bacterial-sensing Toll-like receptors, two composite indices were calculated for each breed: a viral-sensing receptor index (VRI) and a bacterial-sensing receptor index (BRI). Both were defined as unweighted arithmetic means of the relative gene expression of three receptors. Because this approach assigns equal weight to each receptor, VRI and BRI are referred to throughout as exploratory equal-weight indices. They summarize transcript abundance of selected receptors and were not designed or validated as measures of functional antiviral or antibacterial capacity.

### Exploratory equal-weight Viral-sensing Receptor Index (VRI)

The breed wise VRI(VRI_b_) was calculated using the expression profiles of TLR3, TLR7, and TLR21, which are associated with viral nucleic-acid sensing and antiviral innate immune responses. In chickens, TLR3 recognizes double-stranded RNA (dsRNA), whereas TLR7 recognizes single-stranded RNA (ssRNA). TLR21, the avian functional homologue of mammalian TLR9, recognizes unmethylated CpG-containing DNA and may contribute to innate immune responses against DNA viruses and other microbial nucleic acids (Brownlie and Allan, 2011; Chen et al., 2013; Brownlie et al., 2009; Ruan et al., 2014). Accordingly, VRIb was defined as a study-specific composite index representing the relative expression of TLRs associated with antiviral and viral nucleic-acid recognition, rather than an exclusive measure of viral ligand specificity.Based on the extent of efficiency of TLR binding with NDV G-II strain, we have proposed breed wise viral recognition index (VRI_b_) by considering specific TLRs invovled in viral recognition.

$$\text{VR}I_{b}=\frac{\text{TLR}3+\text{TLR}7+\text{TLR}21}{3}$$

### Exploratory equal-weight Bacterial-sensing Receptor Index (BRI)

The BRIb was calculated using the expression profiles of TLR2A, TLR2B, TLR4, and TLR5, which are primarily associated with the recognition of bacterialS pathogen-associated molecular patterns. The chicken TLR2 subfamily recognizes bacterial lipoproteins and peptidoglycan-associated components, TLR4 recognizes bacterial lipopolysaccharide (LPS), and TLR5 recognizes bacterial flagellin (Brownlie and Allan, 2011; Higuchi et al., 2008; Keestra et al., 2008). Higher the values were considered to be involved in viral and bacterial recognition to a better extent.

$$\text{BR}I_{b}=\frac{\text{TLR}2A+\text{TLR}4+\text{TLR}5}{3}$$

### Sensitivity analyses of index weighting

To evaluate whether the assumption of equal receptor contributions influenced the breed comparisons, two alternative indices were calculated. For these analyses, each TLR variable was first standardized (z-score) across all birds so that receptors measured on different scales contributed comparably. As a stricter test of the contribution of TLR21, a VRI restricted to the receptors most directly involved in RNA sensing was calculated as the mean of standardized TLR3 and TLR7 values.

Weights were derived from the loadings (eigenvector coefficients) of the first principal component. For each index, the PC1 loadings of its three receptors were normalized to sum to 1 and used to compute a weighted sum of the standardized values. The stability of the PC1 loadings was assessed by nonparametric bootstrap resampling of individual birds (1,000 iterations, random seed set to 123). PCA was repeated on each resampled dataset, the sign of PC1 was aligned across iterations, and 95% percentile intervals were calculated for each loading. Because birds were resampled independently of breed, these intervals are intended as a descriptive check of loading stability and not as formal inference. Agreement among index versions was assessed using Spearman rank correlation coefficients based on breed-level mean values (n = 15 breeds; 6 birds per breed), with concordance in breed rankings examined across index versions. Differences among breeds within each index version were evaluated using the Kruskal–Wallis test.

### Immune breadth index

Immune breadth score for each breed was calculated based on how many of TLRs is showing above the overall mean of all TLRs being expressed in a particular breed. Higher the score indicates greater TLR divergence.

### Immune Diversity Score (IDS)

To understand how prepared is each breed, overall, to recognize pathogens, breed wise innate immune index (III_b_) was calculated by TLRs as per following formula. For this purpose 40–ΔCt values of all TLRs in all breeds were standardized using Z-score index. Higher the value indicates the higher preparedness of a breed to pathogen.

$$Z^{2}=\frac{{\left(X-\mu \right)}^{2}}{\sigma }$$

X = breed value; μ = overall mean; σ = standard deviation

$$\text{ID}S_{b}=\frac{\text{TLR}2A+\text{TLR}3+\text{TLR}4+\text{TLR}5+\text{TLR}7+\text{TLR}21}{6}$$

### Shannon TLR diversity index

The true diversity between various TLRs expression within each breed was quantified using Shannon TLR Diversity index as per Ortiz-Burgos (2015). The Shannon Diversity Index measures the evenness or diversity of expression among the different TLRs, irrespective of the total expression level. A higher $H^{\prime }$indicates a more even and diverse distribution of TLR expression across receptors, whereas a lower value indicates that expression is dominated by only a few TLRs.

$$E_{i}=\frac{E-E_{\text{min}}}{E_{\text{min}}-E_{\text{max}}}$$

where $E_{i}$is the normalized (on the basis of minimum to maximum scale) expression of the i-th TLR

$$p_{i}=\frac{E_{i}}{\sum E_{i}}$$

$$H^{\prime }=-\sum \left({p_{i}}^{*}\;\text{ln}\;p_{i}\right)$$

where H′ is the diversity index, p i is the proportion of each species in the sample, and ln p i is the natural logarithm of this proportion (Magurran, 2004; Shannon and Weaver, 1949; Spellerberg and Fedor, 2003).

### In silico protein characterization and protein–protein/RNA-protein docking of TLRs with NDV(G-II) proteins

The amino acid sequences of fusion (F) and hemagglutinin-neuraminidase (HN) proteins and mRNA sequences pertaining to NDV (Genotype VII) and chicken Toll-like receptors (TLR2A, TLR3, TLR4, TLR5, TLR7, and TLR21) were retrieved in FASTA format from the NCBI Protein database (Table 3). The physicochemical properties of the selected proteins, including molecular weight, theoretical isoelectric point (pI), amino acid composition, instability index, aliphatic index, and grand average of hydropathicity (GRAVY), were determined using the ProtParam tool available through the ExPASy server. Secondary structural characteristics, including the relative proportions of α-helices, β-turns, extended strands, and random coils, were predicted using the Self-Optimized Prediction Method with Alignment (SOPMA) server.

**Table 3:** Details of protein accession numbers of TLRs and NDV structural protein sequences used in the study.

Protein	Organism	NCBI ID	PDB ID	AlphaFold Model ID
F	Avian orthoavulavirus 1 (NDV)	AEL75045.1	3MAW	—
HN	Avian orthoavulavirus 1 (NDV)	AEL75044.1	1SUX	—
TLR2A	*Gallus gallus*	ACR26453.1	—	AF-Q9DD78-F1
TLR3	*Gallus gallus*	ACR26363.1	—	AF-C4PCD8-F1
TLR4	*Gallus gallus*	ACR26315.1	—	AF-Q7ZT45-F1

The transmembrane topology of the selected viral and host proteins was predicted using DeepTMHMM. Experimentally determined 3-dimensional structures of the NDV F and HN proteins were retrieved from the Research Collaboratory for Structural Bioinformatics Protein Data Bank (RCSB PDB) in PDB format. As experimentally resolved structures were unavailable for the selected chicken TLRs, predicted structural models of TLR2A, TLR3, TLR4, TLR5, TLR7, and TLR21 were obtained from the AlphaFold Protein Structure Database and I-TASSER. The retrieved and predicted structures were inspected and visualized using PyMOL to assess structural integrity and identify obvious structural inconsistencies prior to molecular docking.

Protein–protein docking was subsequently performed using the PatchDock web server (https://bio3d.cs.huji.ac.il/webserver/patchdock/). Blind docking was employed to identify potential interaction interfaces between the NDV proteins and the selected chicken TLRs without imposing a predefined binding site. Twelve independent docking analyses were performed, comprising F–TLR2A, F–TLR3, F–TLR4, F–TLR5, F–TLR7, F–TLR21, HN–TLR2A, HN–TLR3, HN–TLR4, HN–TLR5, HN–TLR7, and HN–TLR21 complexes. For each docking analysis, the top 5 docking solutions generated by PatchDock were retained for comparative evaluation. The PatchDock score, interface area (Å²), and atomic contact energy (ACE) were recorded for each solution. The mean PatchDock score of the top 5 solutions was calculated for each protein–protein combination and used for comparative assessment of predicted interaction propensity. Higher PatchDock scores were interpreted as indicative of greater geometric shape complementarity between the interacting molecular surfaces. The mean docking scores obtained for the F and HN proteins against the six selected TLRs were subsequently compared. The TLR exhibiting the highest mean PatchDock score for each viral protein was considered to have the highest predicted structural interaction among the receptors evaluated.

To assess potential interactions between NDV RNA secondary and intracellular/endosomal RNA-sensing TLRs, representative RNA fragments corresponding to the F and HN genes were selected. Because full-length F and HN mRNAs are computationally challenging for three-dimensional RNA modelling, representative fragments of approximately 150 nucleotides were used for subsequent structural analysis. RNA secondary structures were predicted using RNAfold version 2.6.3. The predicted RNA structures were subsequently used to generate three-dimensional RNA models with RNAComposer. For RNA–protein docking, the predicted three-dimensional RNA structures of the representative F and HN mRNA fragments were docked independently with chicken TLR3 and TLR7 structures obtained from AlphaFold using the HDOCK server. The top-ranked docking complexes were selected for comparative analysis and visualized using PyMOL. Docking scores and confidence scores were used to compare the predicted interaction strengths between the NDV RNA fragments and the two RNA-sensing TLRs.

### Statistical analysis

The generated data were first evaluated for normality and homogeneity of variance using the Shapiro-Wilk and Levene’s tests, respectively. Data are represented as Mean ± Standard Error (SE) of 6 biological replicates from each breed and were analyzed by one-way ANOVA using IBM SPSS Statistics for Windows software (IBM SPSS 20.0; IBM Corp., Armonk, NY, USA) with Tukey’s post hoc test as multiple comparison test. All the graphs were developed using GraphPad Prism® 8.0. (GraphPad, La Jolla, CA, USA). Values were considered significant and highly significant at 5% and 10% respectively. To assess the biological significance rather than just statistical, effect sizes (ω²) were calculated to understand the magnitude of breed differences beyong P values and to provides a less biased (population-level) estimate.

Multivariate analysis was also conducted, by executing principal component analysis (PCA) to explore the influence breed on TLR expression as primary variable. Metadata such as breed was considered as supplementary variables to highlight individuals in PCA biplots. Treatment groups were included as categorical variables to enhance visualization of clustering patterns. Hierarchically clustered heat map and Pearson’s correlation were constructed, following the approach of Gupta et al. (2022, 2025), to reveal patterns in relative gene expression data sets and potential relationships among response variable respectively.

PCA was performed on the six TLR variables (TLR2A, TLR3, TLR4, TLR5, TLR7, TLR21) after centering and scaling each variable to unit variance, using the prcomp function in R version software 4.4.1 (R Core Team, 2020), utilizing the "ComplexHeatmap" and "FactoMineR" packages. The first two components explained 55.9% and 16.5% of the total variance, respectively. Graphical representation of the data was performed using GraphPad Prism version 10.0 (GraphPad Software, La Jolla, CA, USA). Scatter plots were constructed to depict the relative fold expression of various gene markers in PBMCs across breeds.

## Results

In the present study, we investigated the mRNA expression of TLR2A, TLR3, TLR4, TLR5, TLR7, and TLR21 in PBMCs isolated from 15 different chickens belonging to exotic, fancy, indigenous, and synthetic breeds. A total of 6 biological replicates per breed with two technical qPCR replicates, yielding 12 qPCR reactions per breed were employed for SYBR Green based qPCR analysis. The technical replicate values were averaged before analysis. Selection of stable house-keeping or reference gene is critical in qPCR analysis to obtain reliable results. We compared the suitability of two reference genes, GAPDH and β-actin, using different algorithms like GeNorm, BestKeeper©, delta CT and Ref-Finder. Generated Ct values for GAPDH and β-actin expression in the PBMCs from different chicken breeds were in the range of 16.48-20.26 and 10.48-18.13, respectively. Different software algorithms including GeNorm, Bestkeeper©, delta CT and RefFinder suggested the more stable expression of GAPDH than β-actin in the chicken PBMCs (Table 4). Therefore, GAPDH was selected as the reference gene for further qPCR experiments.

**Table 4:** Reference genes expression stability values as calculated by different algorithms.

Genes	Geomean value	Bestkeeper	GeNorm	Delta CT	RefFinder rank
GAPDH	1.189	0.826	1.74	1.74	1
β-actin	1.414	1.216	1.74	1.74	2

Correlation analysis of TLRs gene expression revealed distinct co-expression patterns (Fig. 1). Strong and significant positive correlations were observed among TLR21, TLR2A, TLR3, TLR5, and TLR7, suggesting coordinated regulation of innate immune signalling pathways following ND vaccination. This suggests the existence of a tightly integrated innate immune network rather than independent receptor activation pathways. The strongest positive correlations were observed among TLR3, TLR5, and TLR7, suggesting that these receptors may be co-regulated during vaccine-induced immune activation. TLR3 and TLR7, both involved in viral nucleic acid recognition, exhibited particularly strong associations, reflecting their complementary roles in antiviral defense mechanisms. Moderate-to-strong positive correlations were also observed between TLR2A and TLR5, TLR2A and TLR7, and TLR3 and TLR5, indicating substantial overlap in receptor activation patterns across chicken populations. Similarly, TLR21 displayed positive correlations with several TLRs, although the magnitude of these relationships was generally lower than those observed among TLR3, TLR5, and TLR7. In contrast, TLR4 showed weaker and fewer significant associations with other TLRs, indicating a distinct or partially independent regulatory pattern. The absence of negative correlations is noteworthy and indicates that none of the investigated receptors exhibit antagonistic expression patterns. Instead, birds exhibiting elevated expression of one receptor generally display increased expression of several other receptors, reflecting broad activation of innate immune pathways.

**Fig. 1:**
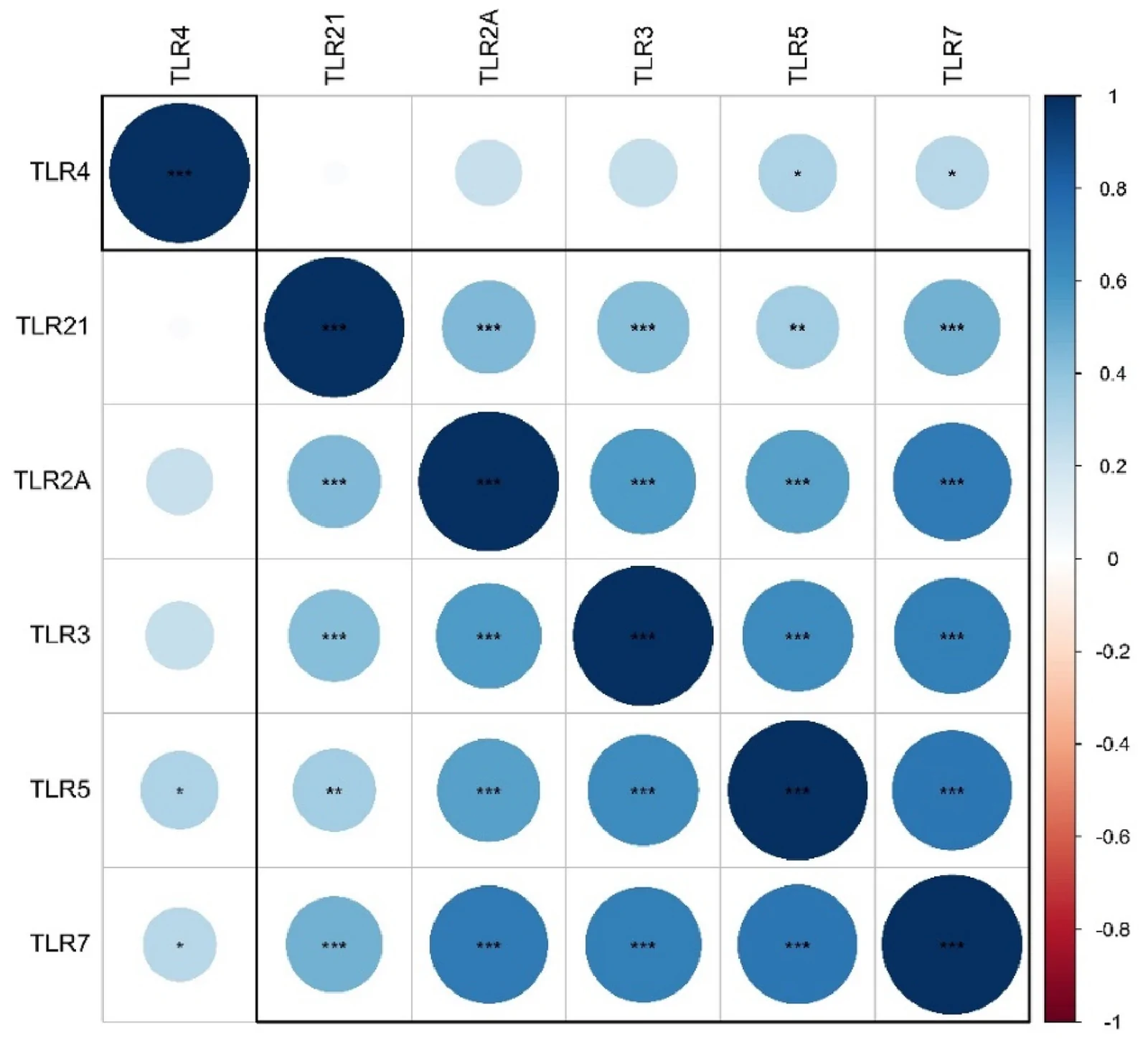
Correlation matrix of Toll-like receptor (TLR) gene expression across different chicken breeds. Bubble size and color intensity represent the strength of the correlation (Pearson’s r), with blue indicating positive correlations and red indicating negative correlations. Statistical significance is denoted by asterisks (*p < 0.05, **p < 0.01, ***p < 0.001).

Principal component analysis was performed to evaluate breed-wise variation in TLR gene expression profiles (Fig. 2). Although substantial overlap among confidence ellipses was observed, distinct breed-specific separation was evident. Notably, the SFLB breed was clearly segregated in the upper-left quadrant of the PCA plot. In contrast, the WFRZ and KN breeds clustered in the lower-right quadrant, indicating suggesting substantial divergence of TLR expression patterns from those of other breeds. Furthermore, the CSH breed showed a strong positive association with Dim1, driven primarily by high loadings for TLR7, TLR5, TLR3 and TLR2A, suggesting coordinated regulation of these innate immune receptors. The variables plot revealed that TLR3, TLR5, TLR7, TLR2A, and TLR21 loaded strongly and positively on Dim1, whereas TLR4 was more strongly associated with Dim2. The PCA score plot revealed clear breed-dependent clustering patterns. Several synthetic and crossbred populations, including AR, BCSFL, BCSML, CBD, and CSH, clustered predominantly on the positive side of PC1, indicating relatively similar expression profiles characterized by elevated expression of multiple TLR genes. In contrast, RIR, NCB, and SFLB were positioned toward the negative side of PC1, suggesting distinct transcriptional responses compared with the synthetic populations.

**Fig. 2:**
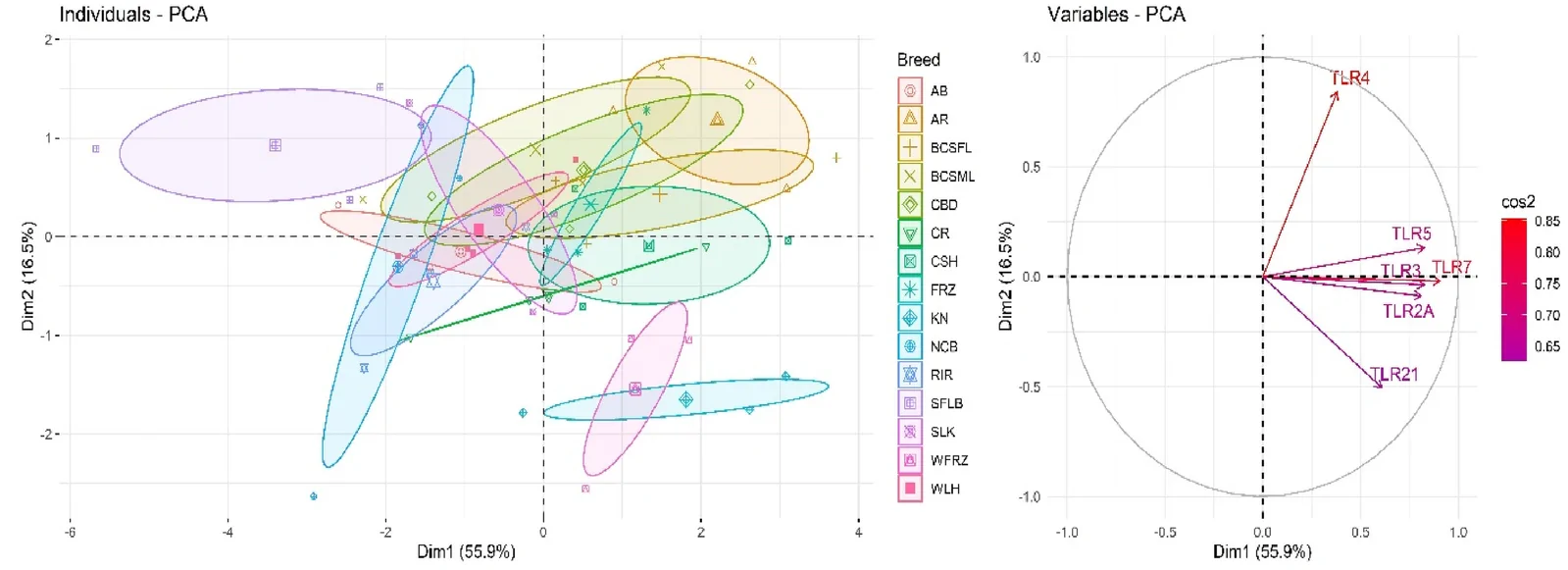
Principal component analysis (PCA) of Toll-like receptor (TLR) gene expression across different chicken breeds under normal conditions. The individuals plot (left) shows the distribution of samples according to breed, with ellipses representing the 95% confidence intervals. The variables plot (right) illustrates the contribution and direction of individual TLR genes to the principal components. Dim1 and Dim2 explain 55.9% and 16.5% of the total variance, respectively. Arrows indicate the loading strength and orientation of each gene, with longer arrows reflecting higher contributions to sample discrimination.

Sample-level clustering revealed heterogeneity among chicken lines, with certain lines showing consistently elevated expression of multiple TLRs, while others exhibited globally reduced TLR expression. With no certain gene expression recognized across the various breeds (Fig. 3). The heat map strongly corroborates the PCA findings with TLR3, TLR5, TLR2A, TLR7, TLR21 as the principal drivers of breed separation. The heat map demonstrated similar pattern by showing coordinated upregulation of these genes in the clusters containing indigenous and synthetic populations. Likewise, TLR4 formed a separate branch in the heat map and contributed primarily to PC2 in PCA, confirming its relatively independent regulation. Dominated by SFLB, BCSML, AB and NCB, Cluster 1 (Far Right) represents comparatively expression of all the TLRs studied indicating lower innate immune activation following vaccination. Cluster 3 inlcudes BCSML, BCSFL, WLH, CBD, SLK, FRZ, CR with the expression pattern characterized by moderate TLR3, TLR4, TLR7 expression while the TLR21 is variable. Cluster 2 contains WLH, RIR, AB, NCB with reduced TLR7 expression. Cluster 4 with KN, WFRZ and CSH exhibiting robust activation of antiviral recognition pathways as indicated by elevated TLR7 and TLR21 gene expression. Cluster 5 AR, BCSFL, BCSML, CBD are mostly immunologically responsive with consist expression of TLR7.

**Fig. 3:**
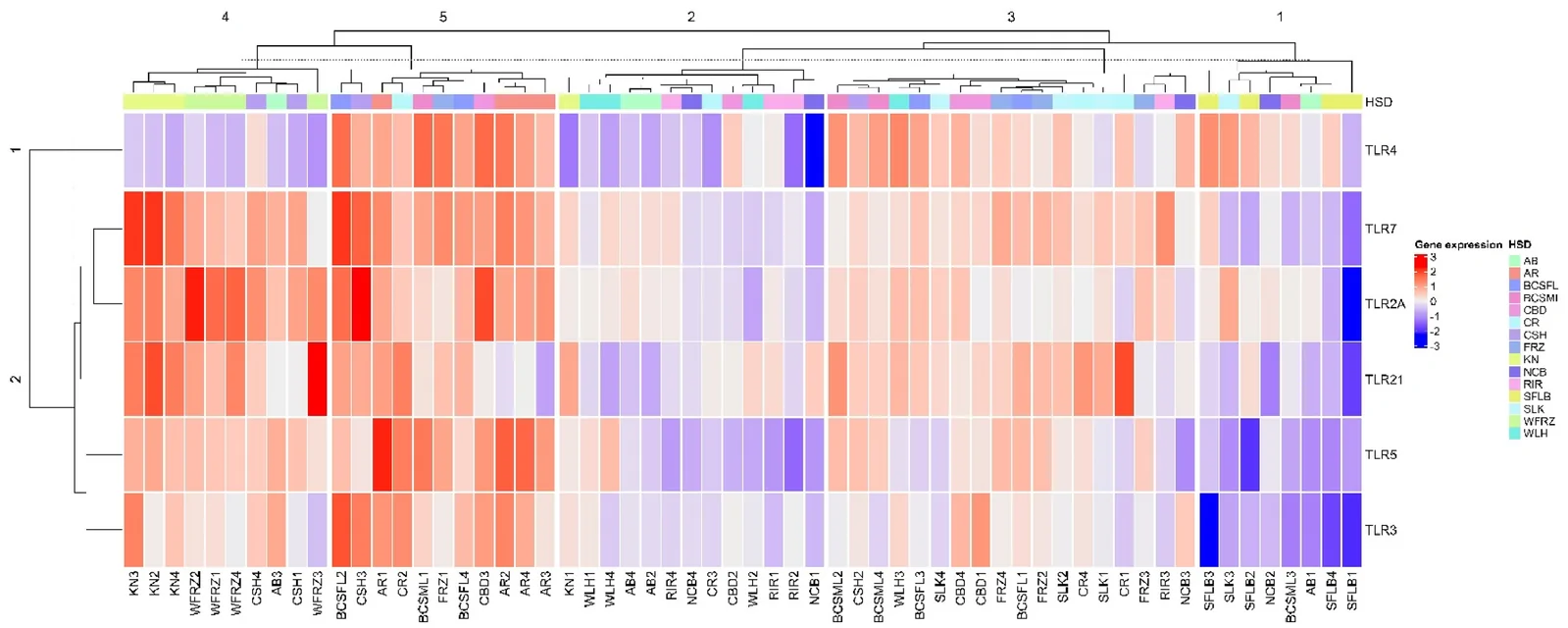
Hierarchical clustering heatmap of Toll-like receptor (TLR) gene expression across chicken genetic lines. Rows represent TLR genes (TLR3, TLR5, TLR21, TLR2A, TLR7, and TLR4), and columns represent individual chicken lines. Gene expression values are shown in red (higher expression) and blue (lower expression). The column dendrogram classified birds into five major expression clusters. These clusters generally correspond to breed-specific immune response patterns rather than random grouping.

### Expression of bacterial sensing Toll-like receptors (TLR2A, TLR4 and TLR5)

Table 5 shows the comprehensive expression values (40–ΔCt) of various TLR genes in PBMC of different chicken breeds. The expression of bacterial recognition receptors differed significantly following ND vaccination (P < 0.001), demonstrating considerable breed-dependent variation in early innate immune activation. Overall, indigenous and several synthetic populations exhibited relatively greater activation of bacterial sensing pathways than the exotic breeds.

**Table 5:** TLRs gene expression in PBMCs of different chicken breeds (Means±SE).

	TLR2A	TLR3	TLR4	TLR5	TLR7	TLR21
WLHC	34.26^d^±0.11	33.17^c^±0.19	33.87^c^±0.08	33.19±0.17^d^	32.69^e^±0.19	32.18^d^±0.47
WLH	36.23^bcd^±0.29	36.67^a^±0.15	35.85^abc^±0.47	34.30^bcd^±0.30	35.17^bcd^±0.16	33.04^bcd^±0.34
RIR	36.31^bcd^±0.13	36.36^a^±0.11	35.40^bc^±0.33	33.41^cd^±0.23	35.44^abcd^±0.34	32.93^cd^±0.13
CR	36.23^bcd^±0.17	36.84^a^±0.38	35.91^abc^±0.38	34.54^bcd^±0.42	35.41^abcd^±0.16	34.19^ab^±0.39
AR	37.26^abcd^±0.05	37.56^a^±0.25	36.89^a^±0.17	35.99^a^±0.22	36.33^ab^±0.14	33.05^bcd^±0.38
AB	36.49^bcd^±0.11	36.51^a^±0.42	35.37^bc^±0.14	34.10^bcd^±0.39	35.37^abcd^±0.25	32.57^d^±0.17
KN	37.17^abcd^±0.30	37.22^a^±0.29	35.13^b^±0.18	34.92^ab^±0.17	36.69^a^±0.41	34.48^a^±0.20
NCB	36.00^cd^±0.16	36.44^a^±0.25	35.39^bc^±0.69	33.59^bcd^±0.21	34.91^cd^±0.17	32.81^cd^±0.35
CSH	37.47^ab^±0.38	37.17^a^±0.33	36.11^abc^±0.35	34.75^abc^±0.10	36.17^abc^±0.28	33.50^abcd^±0.17
CBD	36.80^abcd^±0.49	37.40^a^±0.25	36.63^a^±0.30	34.51^bcd^±0.36	35.51^abcd^±0.28	33.20^abc^d±0.07
BCSML	36.45^bcd^±0.05	36.37^a^±0.33	36.89^a^±0.27	34.75^abc^±0.47	35.29^bcd^±0.33	33.47^abc^±0.27
BCSFL	35.52^d^±0.55	34.86^b^±0.42	36.28^ab^±0.39	33.18^d^±0.31	34.57^d^±0.38	32.40^d^±0.44
SFLB	36.97^abcd^±0.33	37.36^a^±0.42	36.82^a^±0.26	34.64^bc^±0.23	36.27^abc^±0.35	33.83^abc^±0.09
FRZ	36.50^bcd^±0.12	36.72^a^±0.03	36.32^ab^±0.37	34.97^ab^±0.10	36.05^abc^±0.14	33.44^abc^±0.16
WFRZ	37.95^a^±0.19	36.63^a^±0.16	35.24^bc^±0.14	34.76^abc^±0.09	35.69^abcd^±0.19	34.39^ab^±0.38
SLK	36.65^abcd^±0.17	36.37^a^±0.17	36.34^ab^±0.26	33.95^bcd^±0.25	35.35^abcd^±0.27	33.34^abcd^±0.32
P value	0.000	0.000	0.000	0.000	0.000	0.000

^abcd^Means bearing different superscripts within column differ significantly. P value < 0.001 differ significnalty higher.

Among the indigenous breeds, AR consistently showed one of the highest expression levels for TLR2A, TLR4 and TLR5, indicating broad activation of bacterial pathogen recognition mechanisms. CR also maintained balanced expression across all three receptors, whereas KN exhibited elevated TLR2A and TLR5 expression with moderate TLR4 expression. In contrast, NCB displayed comparatively lower expression of TLR2A and TLR5 than the remaining indigenous breeds. Within the synthetic populations, CSH and CBD demonstrated consistently high expression of all three bacterial recognition receptors, whereas BCSML exhibited particularly elevated TLR4 expression. Conversely, BCSFL showed significantly lower expression of TLR2A and TLR5 despite maintaining moderate TLR4 expression. SFLB exhibited relatively higher expression across all three receptors compared with BCSFL. Among the fancy breeds, WFRZ exhibited the highest TLR2A expression among all evaluated genetic groups, while FRZ maintained comparatively higher TLR5 expression. Silkie (SLK) showed intermediate expression across all bacterial recognition receptors. The exotic layer breeds (WLH and RIR) generally exhibited moderate bacterial receptor expression, with no pronounced activation of any single receptor relative to the indigenous populations.

### Expression of viral sensing Toll-like receptors (TLR3, TLR7 and TLR21)

Marked differences were also observed in the expression of viral recognition receptors among the evaluated chicken populations (P < 0.001), indicating genetic variation in the capacity to recognize viral nucleic acids following ND vaccination. Among the indigenous breeds, KN exhibited one of the strongest antiviral receptor signatures, characterized by significantly higher expression of TLR7 and TLR21 together with elevated TLR3 expression. Aseel Red also demonstrated high expression of all three viral sensing receptors, while CR maintained relatively balanced expression across the antiviral receptor repertoire. Aseel Black exhibited moderate expression of TLR3 and TLR7 but comparatively lower TLR21 expression. Nicobari consistently showed lower expression of TLR7 and TLR21 than the remaining indigenous breeds. Among the synthetic populations, CSH, CBD, and SFLB exhibited balanced activation of TLR3, TLR7 and TLR21, whereas BCSFL consistently recorded comparatively lower expression of all three viral recognition receptors. Within the fancy breeds, FRZ exhibited relatively high TLR7 expression, whereas WFRZ showed comparatively higher TLR21 expression. Silkie maintained intermediate expression across all viral recognition receptors. The exotic breeds (WLH and RIR) demonstrated moderate expression of TLR3 and TLR7 with comparatively lower TLR21 expression, indicating moderate activation of viral sensing pathways following vaccination.

To test whether equal weighting influenced the breed ranking, VRI was recalculated using PC1 loadings as weights (TLR3 = 0.353, TLR7 = 0.387, TLR21 = 0.260) and as an RNA-sensing-only index (TLR3 + TLR7). PC1-weighted VRI gave rankings virtually identical to the equal-weight index (Spearman ρ = 0.996; only CR and FRZ exchanged adjacent positions). The RNA-only index was also strongly concordant (ρ = 0.896); the highest-ranked breeds (KN, AR, BCSFL, CSH) and the lowest-ranked breeds (NCB, SFLB) were unchanged, whereas intermediate breeds shifted by up to four positions (Supplementary Table 1-3, Supplementary Fig. 1-2). Breed differences were significant for all three versions (Kruskal-Wallis, p ≤ 0.001).

### Breed-specific innate immune architecture following ND vaccination

To obtain a systems-level understanding of innate immune responsiveness, receptor expression profiles were integrated into composite immune indices representing bacterial recognition, viral recognition, immune diversity and overall receptor distribution. Regarding immune diversity scores (IDS), the indigenous breeds AR and KN exhibited significantly higher TLR diversity (P < 0.001), followed by intermediate scores in synthetic populations (BCSFL, CSH, CBD, and WFRZ), while the lowest scores were observed in SFLB, SLK, NCB, and AB. The VRI demonstrated marked inter-breed variability, with K exhibiting the highest viral TLR score (1.16), followed by CBD (0.75), AR (0.54), CSH (0.49), and WFRZ (0.43). AR exhibited the highest BRI (1.20), followed by CBD (0.52) and CSH (0.50), This suggests a breed-specific capacity to respond to diverse pathogen types, with KN showing greater efficiency in viral responses and AR in bacterial pathogen defense (Fig. 5).

Despite these differences, the Shannon TLR Diversity Index remained remarkably conserved across breeds, indicating that ND vaccination elicited relatively balanced utilization of the six evaluated TLRs (Table 6). One-way ANOVA revealed substantial differences (P < 0.01) in Shannon diversity index among the 15 chicken breeds (F₁₄,₄₅ = 5.85). Breed accounted for a large proportion of the observed variation (ω² = 0.531), indicating that genetic background explained approximately 53–65% of the variation in TLR diversity. Most breeds exhibited comparable and relatively high TLR diversity values (1.68–1.77), indicating a balanced distribution of ND vaccine–induced TLR expression. The highest indices were observed in CR, KN, FRZ and WFRZ, with indigenous breeds generally showing higher diversity than fancy, synthetic, and exotic lines. In contrast, the SFLB line exhibited a significantly lower Shannon index (1.42), suggesting a less even TLR expression profile and reduced innate immune diversity, with the response being dominated by a limited subset of TLRs (Fig. 5).

**Table 6:** Shannon and Immune breadth indices of TLRs across various chicken breeds (Means±SE).

	Shannon Index	Immune Breadth Index (designated TLRs)
WLH	1.74^a^ ± 0.01	3 (2/3/4)
RIR	1.72^a^ ± 0.01	5 (2/3/4/7)
CR	1.77^a^ ± 0.00	4 (2/3/4/7)
AR	1.73^a^ ± 0.01	4 (2/3/4/7)
AB	1.73^a^ ± 0.01	4 (2/3/4/7)
KN	1.76^a^ ± 0.00	3 (2/3/4/7)
NCB	1.68^a^ ± 0.04	4 (2/3/4/7)
CSH	1.75^a^ ± 0.01	4 (2/3/4/7)
CBD	1.74^a^ ± 0.00	3 (2/3/4)
BCSML	1.74^a^ ± 0.01	3 (2/3/4)
BCSFL	1.76^a^ ± 0.00	4 (2/3/4/7)
SFLB	1.42^b^ ± 0.14	3 (2/3/7)
FRZ	1.76^a^ ± 0.00	4 (2/3/4/7)
WFRZ	1.76^a^ ± 0.00	2 (2/3)
SLK	1.73^a^ ± 0.04	4 (2/3/4/7)
P value	0.000	-

^ab^Means bearing different superscripts within column differs highly significantly (P < 0.01).

The Immune Breadth Index (IBI), calculated based on the number of TLRs exhibiting balanced vaccine-induced expression, revealed considerable variation among the evaluated chicken genetic groups. The majority of breeds (10/15) demonstrated a broad innate immune recognition profile with an IBI of 4, characterized by the coordinated involvement of TLR2A, TLR3, TLR4, and TLR7 in response to ND vaccination. These included RIR, CR, AR, AB, NCB, CSH, BCSFL, FRZ, and SLK, indicating a comprehensive pattern of pathogen recognition. A moderate immune breadth (IBI = 3) was observed in WLH, KN, CBD, and BCSML, where balanced expression was restricted to TLR2A, TLR3, and TLR4, suggesting comparatively narrower activation of innate immune receptors. In contrast, WFRZ exhibited the lowest immune breadth (IBI = 2), with balanced expression limited to TLR2A and TLR3, indicating the most restricted pattern of innate immune receptor engagement among all evaluated breeds.

### Relative fold change expression of TLRs in different breeds

The relative log_2_ FC mRNA expression of toll-like receptors (TLR2A, TLR3, TLR4, TLR5, TLR7, and TLR21) differed significantly among chicken breeds (Fig. 4). Distinct breed-specific expression profiles were observed for each TLR, indicating substantial genetic variability in innate immune sensing pathways. Bars sharing different superscript letters indicate statistically significant differences (P < 0.05).

**Fig. 4:**
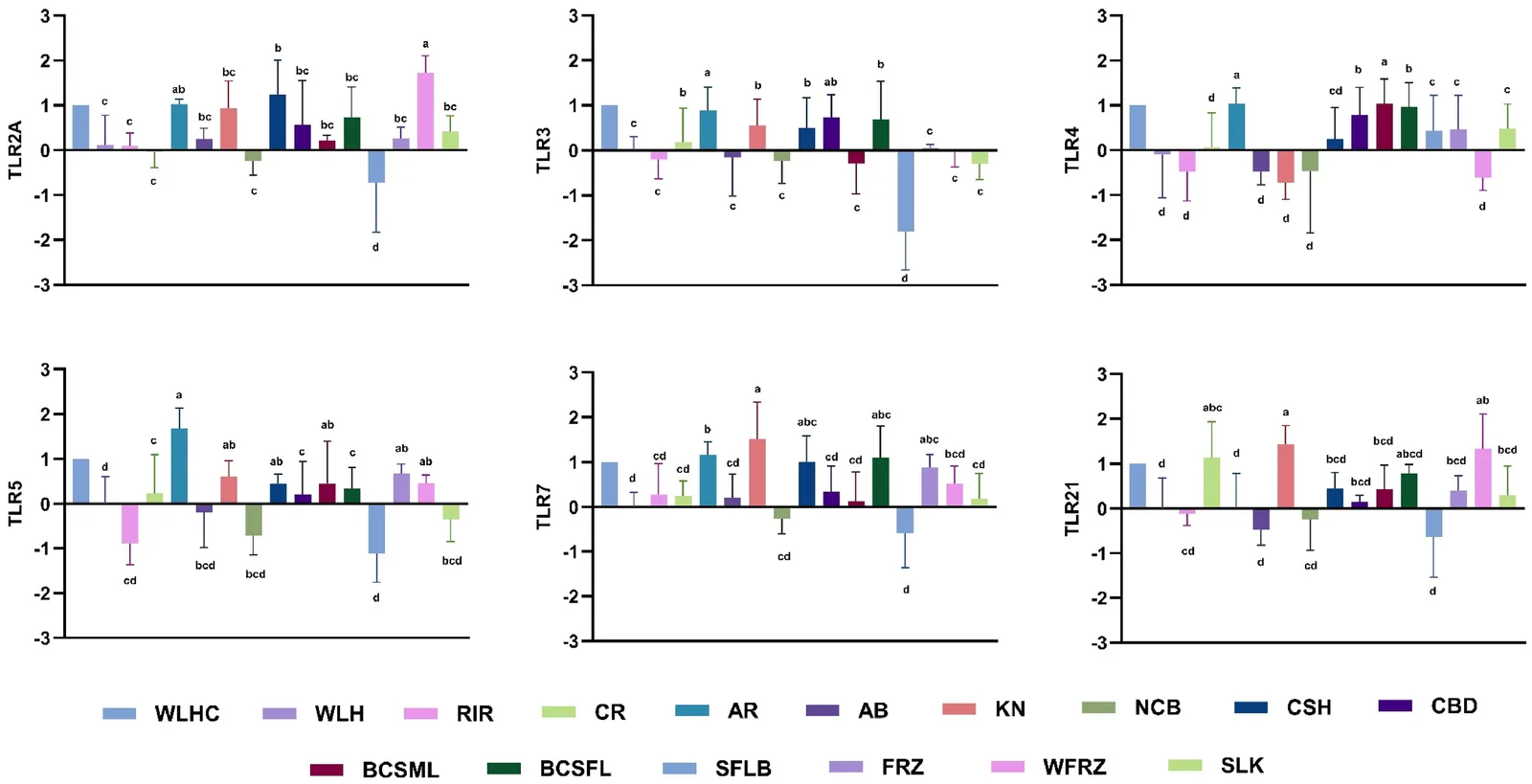
Relative Log_2_ fold chain (FC) expression of various Toll-like receptor (TLR) genes (from left to right) TLR2A, TLR3, TLR4, TLR5, TLR7 and TLR21 in different chicken breeds or variety (exotic, indigenous, fancy and crosses). Bars bearing different superscripts differ significantly (p < 0.01). Each colour bar graph corresponds to a breed. One way ANOVA results across different chicken breeds revealed a significant difference with p-values 0.00002, 0.00002, 0.00142, 0.00001, 0.00007, 0.00003 and ω² values of 0.477, 0.480, 0.342, 0.544, 0.443, 0.467 for TLR2A, TLR3, TLR4, TLR5, TLR7, TLR21 respectively. WLHC was considered as unvaccinated control group to which the fold chain was calculated for all the ND vaccinated breeds.

TLR2A expression showed marked variation across breeds. Indigenous and locally adapted breeds such as AR, KN, and BCSFL exhibited significantly higher expression compared with commercial or exotic breeds. WFRZ exhibited a strong upregulation of TLR2A expression, significantly higher than all other breeds. In contrast, SFLB showed a pronounced downregulation of TLR2A. Moderate expression levels were observed in CBD, FRZ, and AB, while CR and NCB showed relatively lower expression. Expression of TLR3, a receptor associated with viral double-stranded RNA recognition, was highest in AR, KN, CBD, and BCSFL. SFLB exhibited a strong suppression of TLR3 expression, significantly lower than all other breeds. Most remaining breeds clustered around baseline or slightly positive expression, indicating moderate antiviral sensing capacity. TLR4 expression differed significantly among breeds, with AR, CBD, BCSML, and FRZ showing elevated levels. RIR, AB, KN, NCB, and SLK exhibited significantly reduced expression. The wide spread of values suggests breed-dependent variation in responsiveness to lipopolysaccharide (LPS) and Gram-negative bacterial components.

TLR5 expression was highest in AR, followed by FRZ, and KN. Strong downregulation was observed in RIR, NCB, and SFLB. Several breeds showed intermediate expression, indicating differential capacity for flagellin recognition among genetic lines. TLR7 expression, which is involved in viral single-stranded RNA recognition, was significantly elevated in KN, followed by AR, BCSFL, CSH, FRZ, and WFRZ. SFLB again displayed the lowest expression. The majority of breeds showed positive expression relative to baseline, but with clear stratification into high and moderate responders. TLR21, the avian functional homolog of mammalian TLR9, exhibited highest expression in KN, WFRZ, and CR. Moderate expression was observed in BCSFL, CSH, BCSML, and FRZ, while SFLB, AB, NCB, and RIR showed significantly reduced expression. The pattern suggests breed-specific variation in CpG DNA recognition.

Considering all findings together, PBMCs from Aseel Red showed relatively better TLR expression profile compared to other indigenous breeds (Kadakanath, Aseel Black and Nicobari), exotic breeds (WLH and RIR) as well as fancy breeds (Frizzle and Silkie) and cross breeds. Fancy chicken breed, Frizzle showed a higher expression of TLR3, TLR4, TLR5 and TLR7 in PBMCs as compared to other fancy breeds (Silkie and White Frizzle). In case of cross breeds, the expression of TLR2A, TLR3, TLR4, TLR5, TLR7 and TLR21 were higher in PBMCs of CSH, CBD, BCSML, CSH, BCSFL, and CR, respectively in relationship to other cross breeds evaluated in the present study. In exotic breeds of chicken, White Leghorn exhibited a higher expression of TLR3, TLR4, TLR5 and TLR21 than RIR.

### Insilico docking analysis revealed differential geometric based structural interactions of TLRs with NDV F and HN proteins

#### Structural and physicochemical characteristics of NDV proteins and chicken TLRs

The NDV Fusion (F) and hemagglutinin-neuraminidase (HN) proteins comprised 553 and 577 amino acids, respectively, with predicted molecular masses of 58.90 and 63.24 kDa. The theoretical isoelectric points of F and HN were 8.56 and 7.53, respectively. Both viral proteins were predicted to be stable based on their instability indices of 37.64 and 31.66, respectively. The F protein showed a higher predicted aliphatic index (109.17) and a slightly positive GRAVY value (0.193), whereas HN had an aliphatic index of 81.09 and a negative GRAVY value (−0.164). The chicken TLR proteins ranged from 793 to 1,059 amino acids in length, with predicted molecular masses ranging from 90.77 to 122.27 kDa. Their theoretical pI values ranged from 6.29 to 9.02. The predicted secondary structures of the TLRs were predominantly composed of α-helices and random coils. α-Helical content ranged from 47.92 to 53.81%, whereas random-coil content ranged from 36.79 to 42.12%. In comparison, the F protein comprised 44.30% α-helices, 19.71% extended strands and 35.99% random coils, while HN comprised 18.72% α-helices, 24.26% extended strands and 57.02% random coils. DeepTMHMM predicted a single transmembrane helix in each of the six chicken TLRs. The predicted transmembrane regions occurred near the C-terminal region of the receptors, consistent with their predicted membrane-associated topology. The corresponding signal peptide and extracellular regions were also identified for the TLRs with signal sequences. The detailed structural and physicochemical characteristics of NDV and TLR proteins were shown in Supplementary Table 4.

#### Protein–protein docking of NDV F and HN proteins with chicken TLRs

Blind protein–protein docking was performed between the NDV F and HN proteins and TLR2A, TLR3, TLR4, TLR5, TLR7 and TLR21. The docking analysis revealed receptor-specific differences in the predicted structural complementarity of the two viral proteins (Table 7 and Fig. 6, 7). Among the evaluated F protein–TLR complexes, the highest PatchDock score was observed for the F–TLR5 complex, with a score of 22,798.0. For the HN protein, the highest PatchDock score was observed for the HN–TLR7 complex, with a score of 22,698.0. Thus, based on the PatchDock score, F–TLR5 and HN–TLR7 represented the highest-scoring protein–protein docking combinations identified in the analysis. These results indicated greater predicted geometric complementarity of the F protein with TLR5 and of the HN protein with TLR7 compared with the other evaluated receptor combinations. The docking scores were used for relative comparison of the predicted complexes and were not considered measures of experimentally determined binding affinity.

**Table 7:** Comparative PatchDock Scores (PDS) and Atomic Contact Energy (ACE) of TLRs with NDV proteins.

Protein	TLR2A	TLR3	TLR4	TLR5	TLR7	TLR21
PD score						
Fusion	20142	18373.6	19898.4	22798	17596.8	19651.2
Haemagglutunin neuraminidase	16295.6	18304	17770	17335.6	22698	17931.2
ACE score						
Fusion	−140.38	−30	14.29	96.39	1.19	−408.93
Haemagglutunin neuraminidase	270.37	248.43	167.97	417.54	218.96	394.85

The corresponding Atomic Contact Energy (ACE) values varied across receptor–ligand combinations. For F, the ACE values ranged from −408.93 to 96.39, with the most negative value observed for the F–TLR21 complex. For HN, ACE values ranged from 167.97 to 417.54, with the lowest value observed for HN–TLR4. The differences between PatchDock and ACE rankings indicated that geometric docking scores and predicted energetic parameters did not identify identical top-ranked complexes.

#### Prediction of NDV F and HN mRNA secondary structures

RNAfold analysis demonstrated extensive secondary-structure formation in the representative RNA sequences corresponding to the NDV F and HN genes. The thermodynamic ensemble free energies were −470.48 kcal/mol for F RNA and −529.17 kcal/mol for HN RNA. The minimum free energies associated with the centroid structures were −278.60 and −343.40 kcal/mol for F and HN RNA, respectively. The predicted ensemble diversity was 503.69 for F RNA and 378.60 for HN RNA. These results indicated that both representative viral RNA regions could adopt structured secondary conformations and were subsequently used for three-dimensional RNA modeling and RNA–protein docking.

#### RNA–protein docking with chicken TLR3 and TLR7

The predicted three-dimensional structures of representative F and HN-derived RNA fragments were docked independently with chicken TLR3 and TLR7 proteins using HDOCK. The top 10 best predicted ranks were depicted in Table 8, 9. Distinct differences were observed in the predicted docking scores among the RNA–TLR complexes (Table 10 and Fig. 8). The F RNA–TLR3 complex showed the most favorable average predicted docking score (−545.12), followed by the HN RNA–TLR3 complex (−476.88). In comparison, the F RNA–TLR7 and HN RNA–TLR7 complexes showed docking scores of −365.45 and −327.00, respectively. All four RNA–protein complexes showed HDOCK confidence scores greater than 0.97. The comparative docking pattern indicated that both F- and HN-derived RNA fragments had more favorable predicted docking scores with TLR3 than with TLR7. Among all evaluated RNA–protein combinations, F RNA–TLR3 exhibited the most favorable predicted interaction, whereas HN RNA–TLR7 showed the least favorable docking score. These findings identify TLR3 as the receptor exhibiting the strongest predicted structural association with both representative NDV RNA fragments in the present computational analysis.

**Table 8:** RNA-Protein docking of NDV F (Fusion) gene mRNA with chicken TLR3 and TLR7 protein.

	F-TLR3				F-TLR7			
	Docking Score	Confidence Score	Ligand rmsd (Å)	Interface residues	Docking Score	Confidence Score	Ligand rmsd (Å)	Interface residues
**1**	−365.45	0.9867	46.62	model_1	−327.00	0.9718	108.98	model_1
**2**	−357.8	0.9846	94.78	model_2	−308.51	0.9597	128.79	model_2
**3**	−345.47	0.9803	67.6	model_3	−307.32	0.9588	93.43	model_3
**4**	−341.08	0.9786	89.24	model_4	−305.05	0.9569	74.93	model_4
**5**	−337.81	0.9772	94.8	model_5	−298.82	0.9515	52.80	model_5
**6**	−337.05	0.9768	67.00	model_6	−293.67	0.9465	87.09	model_6
**7**	−331.24	0.9740	64.52	model_7	−291.33	0.9441	107.42	model_7
**8**	−330.36	0.9736	73.41	model_8	−290.65	0.9434	123.95	model_8
**9**	−328.61	0.9727	66.78	model_9	−290.39	0.9431	120.07	model_9
**10**	−326.66	0.9716	110.89	model_10	−289.98	0.9427	137.60	model_10

**Table 9:** RNA-Protein docking of NDV Haemagglutunin-Neuraminidase (HN) gene mRNA with chicken TLR3 and TLR7 protein.

	HN-TLR3				HN-TLR7			
	Docking Score	Confidence Score	Ligand rmsd (Å)	Interface residues	Docking Score	Confidence Score	Ligand rmsd (Å)	Interface residues
**1**	−327.00	0.9718	108.98	model_1	−476.88	0.9986	82.88	model_1
**2**	−308.51	0.9597	128.79	model_2	−446.91	0.9974	107.86	model_2
**3**	−307.32	0.9588	93.43	model_3	−419.30	0.9954	96.35	model_3
**4**	−305.05	0.9569	74.93	model_4	−417.00	0.9952	123.23	model_4
**5**	−298.82	0.9515	52.8	model_5	−405.91	0.994	122.96	model_5
**6**	−293.67	0.9465	87.09	model_6	−403.41	0.9937	84.43	model_6
**7**	−291.33	0.9441	107.42	model_7	−401.43	0.9935	92.19	model_7
**8**	−290.65	0.9434	123.95	model_8	−395.99	0.9928	109.08	model_8
**9**	−290.39	0.9431	120.07	model_9	−392.70	0.9923	127.68	model_9
**10**	−289.98	0.9427	137.6	model_10	−392.52	0.9922	117.27	model_10

**Table 10:** Predicted molecular interactions between NDV proteins and chicken TLRs based on RNA–protein docking analysis.

RNA ligand	Receptor	HDOCK score	Confidence
F	TLR3	−545.12	>0.97
HN	TLR3	−476.88	>0.97
F	TLR7	−365.45	>0.97
HN	TLR7	−327.00	>0.97

## Discussion

Newcastle disease virus (NDV) remains one of the most important viral pathogens affecting poultry worldwide, and successful vaccine-induced protection depends upon rapid activation of innate immune pathways that subsequently shape adaptive immune responses. Host genetic background is a key determinant of susceptibility and immune responsiveness to NDV. Recent findings from our laboratory revealed that the Indian indigenous Aseel breed displayed superior resilience to virulent NDV (Genotype VII) challenge, characterized by enhanced growth performance, elevated IL-1β expression, and an unaltered serum biochemical profile compared with commercial WLH chickens (Girija et al., 2023a, 2023b). Early innate immune responses to a lentogenic Newcastle disease (ND) vaccine strain were evident within 48 h of administration, as indicated by altered lncRNA expression in the Harderian gland of White Leghorn and Fayoumi chickens (Vanamamalai et al., 2021).

In the present study, differences in the expression profiles of TLR2A, TLR3, TLR4, TLR5, TLR7, and TLR21 were observed among diverse chicken genetic groups at 48 h following ND vaccination, encompassing indigenous, exotic, fancy, and synthetic populations. These differences indicate heterogeneity in TLR-associated innate immune profiles among the genetic groups. Rather than suggesting a simple hierarchical ranking of immune responsiveness, the observed expression patterns may reflect differential representation of pathogen-recognition pathways across genetic backgrounds. However, given the single post-vaccination sampling point and the absence of breed-matched unvaccinated controls, these differences should be interpreted as genetic-group-associated expression patterns following vaccination, rather than definitive evidence of differences in vaccine-induced immune responsiveness or inherent immunogenetic superiority.

The integration of PCA, hierarchical clustering, and correlation analyses demonstrated that vaccine-induced innate immunity is mediated through coordinated receptor networks rather than independent activation of individual TLRs. The PCA provides compelling evidence that genetic background influences not only the magnitude of receptor expression but also the architecture of innate immune signaling pathways, thereby contributing to breed differences in vaccine responsiveness among chicken populations (Neerukonda and Katneni, 2020). The fact that PC1 alone explained 55.9% of the total variance indicates that most of the variation among breeds can be attributed to coordinated differences in TLR expression. Such a high proportion of explained variance suggests a strong genetic influence on innate immune responsiveness. A notable observation is the clustering of several synthetic and crossbred populations (BCSFL, BCSML, CBD, CSH and AR) toward the positive side of PC1. The loading plot indicates that this separation is largely driven by elevated expression of TLR7, TLR5, TLR3, TLR2A, and TLR21, receptors involved in recognition of viral nucleic acids and microbial components. Since NDV is an RNA virus, TLR7 and TLR3 are particularly relevant to vaccine recognition and antiviral signaling. The clustering of these synthetic populations suggests that genetic recombination may have combined favorable immune-associated alleles from multiple parental lines, resulting in enhanced responsiveness of innate immune pathways. The distinct positioning of Aseel and Kadaknath indicates that these breeds possesses a unique innate immune signature. Hierarchical clustering analysis also revealed pronounced breed-dependent variation in TLR expression following Newcastle disease vaccination. At the population level, indigenous and synthetic chicken groups generally clustered together and exhibited elevated expression of multiple TLR genes, particularly TLR7, TLR3, and TLR21, whereas several commercial populations displayed comparatively lower expression profiles. The clustering patterns were highly consistent with PCA results, which identified these same receptors as the major contributors to population divergence.

Elevated VRI expression in breed KN suggests an enhanced capacity for the early recognition of viral PAMPs, including double-stranded and single-stranded RNA, and unmethylated CpG DNA. While higher BRI in AP reflects enhanced recognition of bacterial lipoproteins, lipopolysaccharides, and flagellin. Such enhanced constitutive expression may facilitate more rapid activation of innate immune signalling cascades upon bacterial challenge (Gram +ve and -ve), thereby promoting efficient production of pro-inflammatory cytokines, recruitment of immune effector cells, and subsequent pathogen clearance. Overall, the Immune Breadth Index indicated that most indigenous, synthetic, and fancy chicken populations possess a relatively broad spectrum of TLR engagement following vaccination, whereas certain commercial and selected lines displayed comparatively restricted innate immune receptor diversity. These findings suggest genetic variation in the breadth of innate immune sensing, which may contribute to differential responsiveness to vaccination and disease resistance.

The fancy breeds generally demonstrated intermediate TLR expression patterns. Although their composite immune indices were lower than those of several indigenous breeds, they retained relatively balanced activation of viral and bacterial recognition pathways following ND vaccination. This finding suggests that selection for ornamental traits has not markedly diminished the capacity to mount early innate immune responses, although the magnitude and pattern of receptor activation appear distinct from those of indigenous breeds (Fig. 5). White Leghorn (WLH) and Rhode Island Red (RIR), generally exhibited lower composite TLR expression and reduced bacterial recognition indices indciating long term consequences of selection. Synthetic populations, including CSH, CBD and BCSFL, maintained intermediate to relatively high composite immune indices, reflecting balanced activation of both viral and bacterial recognition pathways. Such populations may therefore represent useful intermediate germplasm for breeding programmes targeting both productivity and disease resilience.

**Fig. 5:**
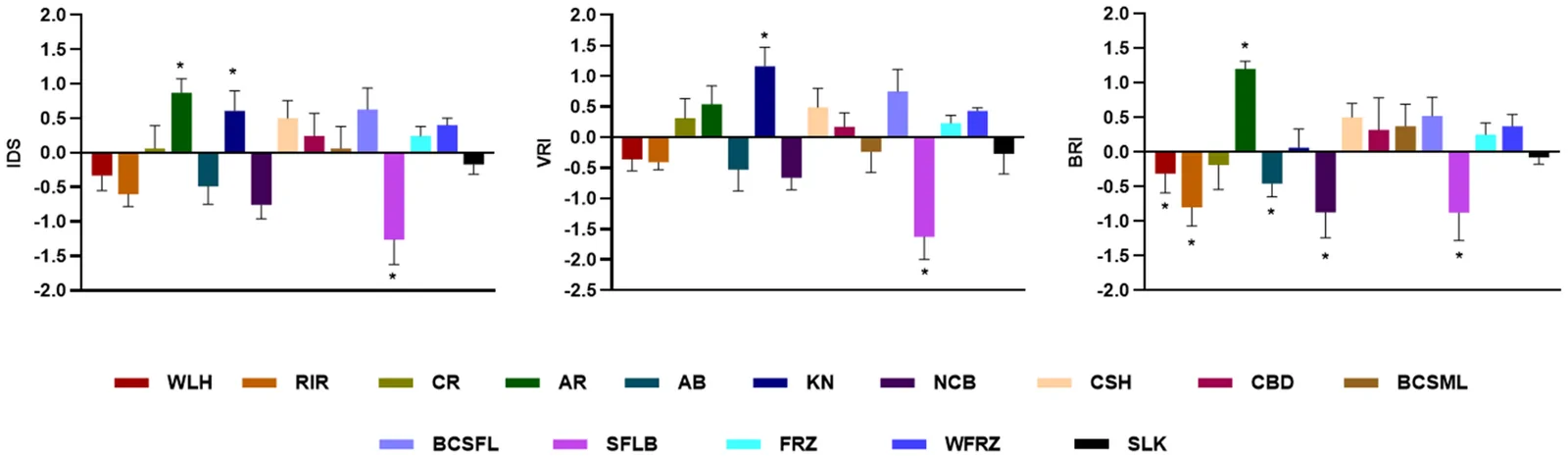
TLR repertoire based innate immune scores across different chicken breeds. Breeds bearing asterisk (*) on column bars differ significantly (p < 0.001). Abbreviations: From left to right: IDS: Immune Diversity Score, VII: Viral Immune Index and BRI: Bacterial Recognition Index.

**Fig. 6:**
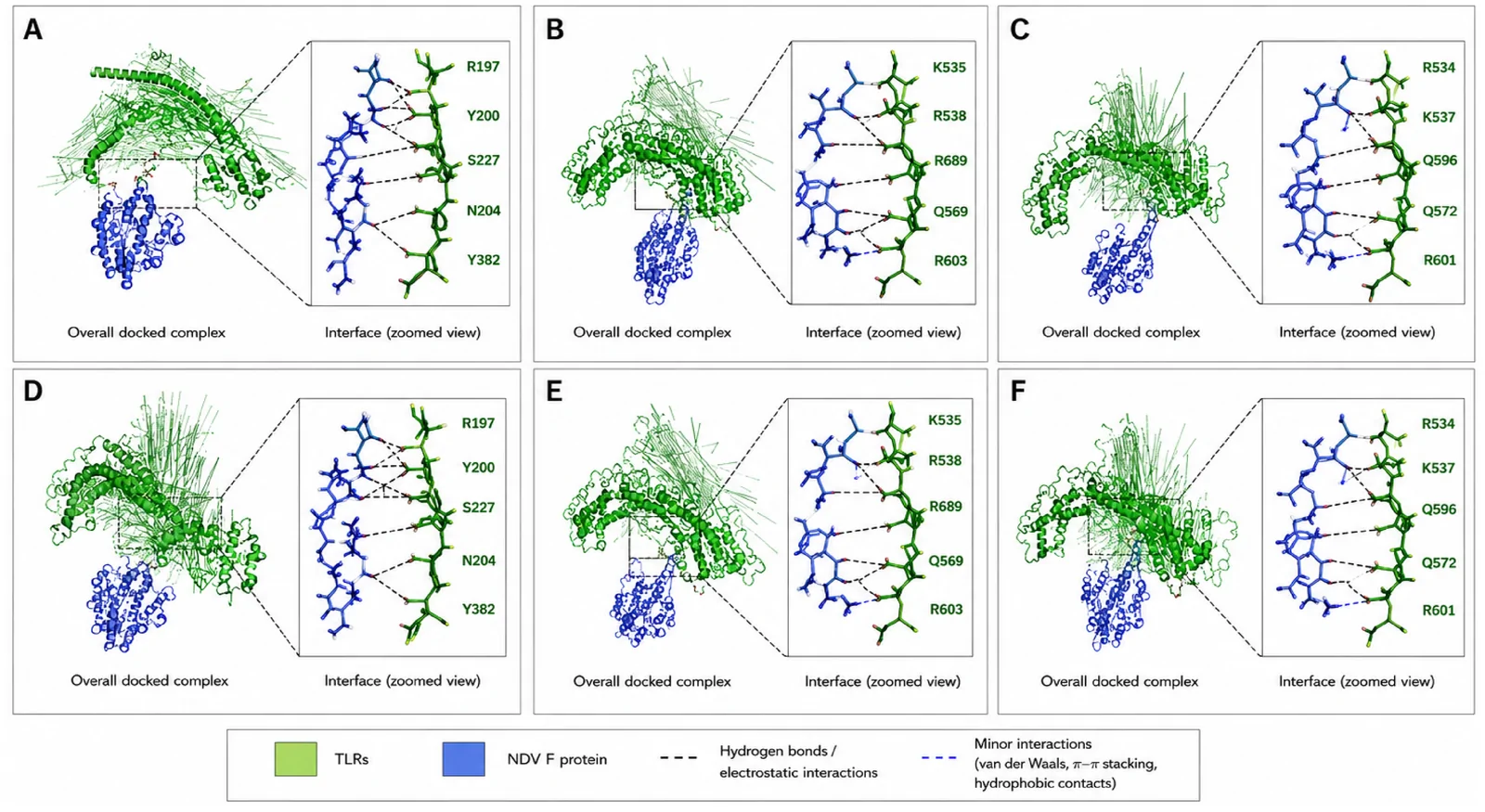
Predicted *in-silico* protein-protein structural interactions of Newcastle disease virus Fusion (F) protein with chicken TLRs. A. F protein with TLR2A, B. F protein with TLR3, C. F protein with TLR4, D. F protein with TLR5, E. F protein with TLR7 and F. F protein with TLR 21. Left panels show overall docked complexes, and right panels show enlarged views of the interacting interface with key residues. The complexes represent computationally predicted structural associations.

**Fig. 7:**
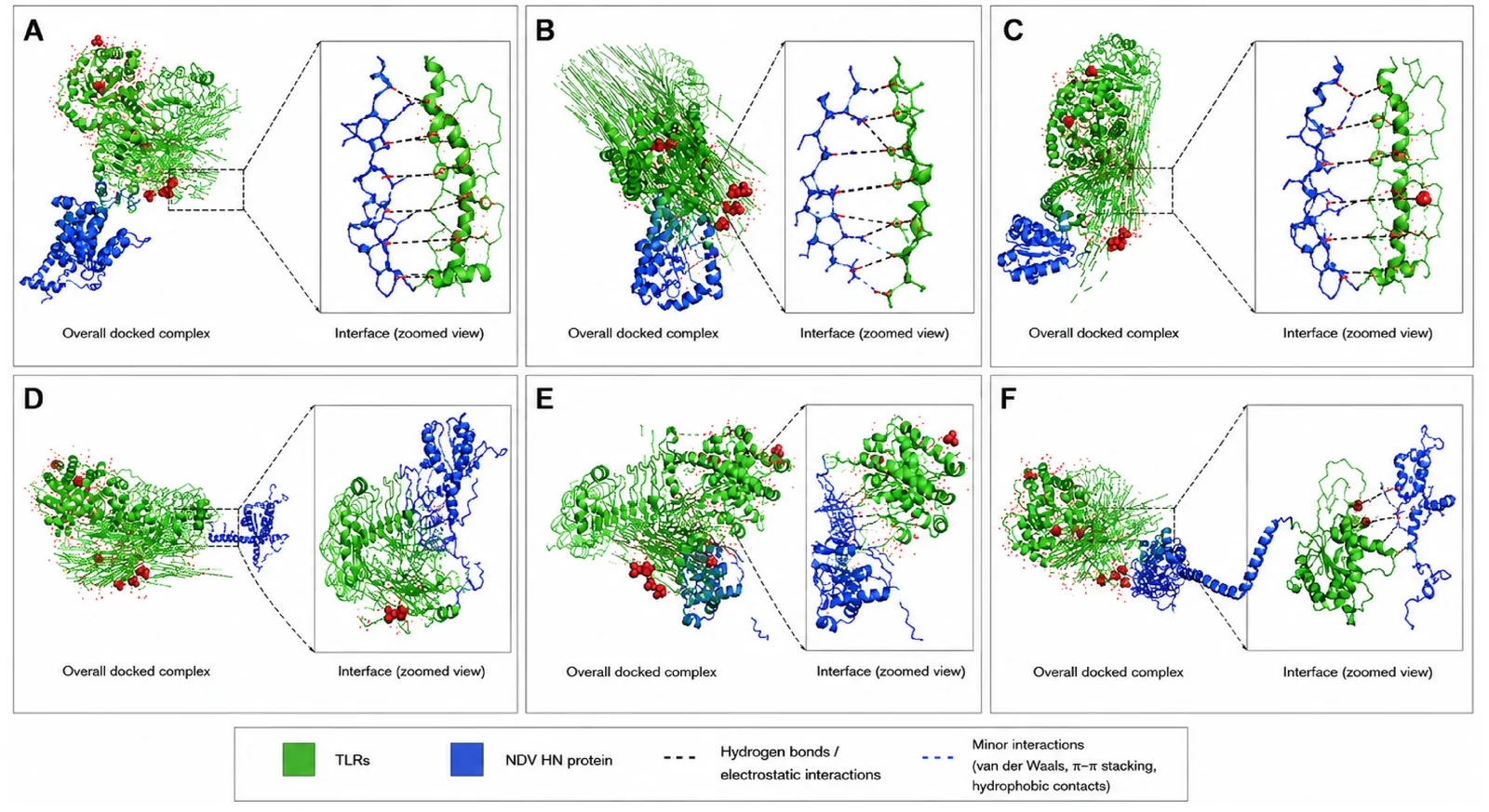
Predicted *in-silico* protein-protein structural interactions of Newcastle disease virus Haemagglutunin-Neuraminidase (HN) protein with chicken TLRs. A. HN protein with TLR2A, B. HN protein with TLR3, C. HN protein with TLR4, D. HN protein with TLR5, E. HN protein with TLR7 and F. HN protein with TLR 21. Left panels show overall docked complexes, and right panels show enlarged views of the interacting interface with key residues.

**Fig. 8:**
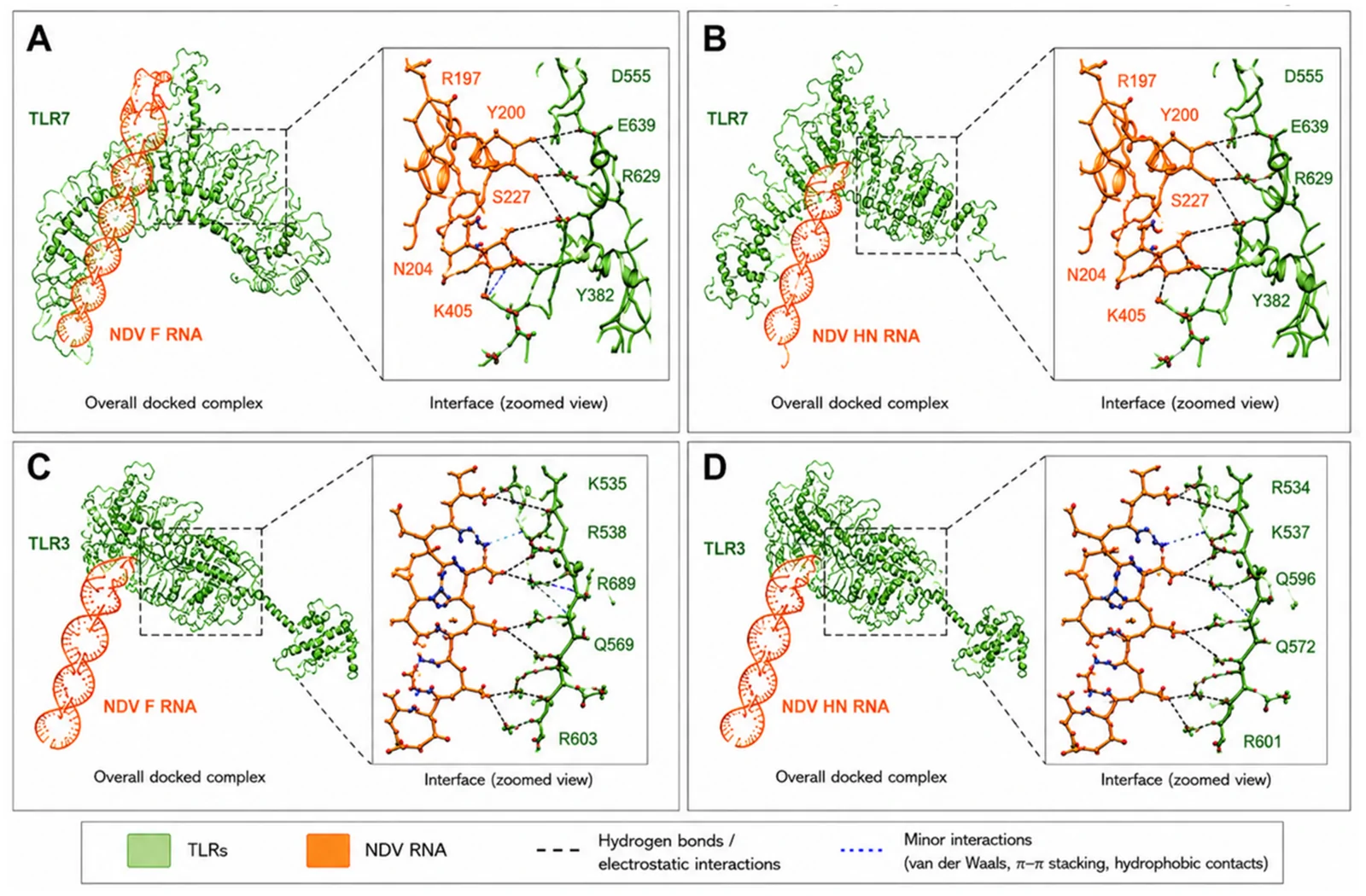
Predicted *in-silico* structural interactions between NDV components and chicken TLRs. Three-dimensional representations of RNA–protein docking complexes are shown for (A) NDV Fusion (F)–TLR7 and (B) Hemagglutinin-neuraminidase (HN)–TLR7. RNA–protein docking complexes are shown for (C) NDV Fusion (F)–TLR3 and (D) Hemagglutinin-neuraminidase (HN)–TLR3. Left panels show overall docked complexes, and right panels show enlarged views of the interacting interface with key residues. The complexes represent computationally predicted structural associations.

Our results likely reflect evolutionary adaptation driven by contrasting selection pressures experienced by different chicken genetic groups. Indigenous chicken breeds have evolved over centuries under extensive scavenging production systems, where continuous exposure to diverse pathogens, fluctuating environmental conditions, and nutritional limitations has favoured natural selection for robust innate immune mechanisms and disease resilience (Lochmiller and Deerenberg, 2000; Rajkumar et al., 2017; Schmid-Hempel, 2003; Yadav et al., 2018). In contrast, commercial and synthetic chicken lines have undergone intensive artificial selection primarily for rapid growth, feed efficiency, egg production, and reproductive performance, potentially reducing the allocation of physiological resources towards constitutive immune functions (Lamont et al., 2002; Emam et al., 2014). According to the theory of evolutionary immune trade-offs, immune defence is energetically expensive, and organisms cannot maximize all fitness-related traits simultaneously. Consequently, investment in production traits is often accompanied by altered investment in immune surveillance and inflammatory responsiveness (Lochmiller and Deerenberg, 2000; Sheldon and Verhulst, 1996).

Our findings support this concept by demonstrating distinct TLR recognition profiles among genetic groups rather than a universally superior immune phenotype. Indigenous breeds generally exhibited broader bacterial and viral recognition capacities together with higher Shannon TLR diversity indices (Fig. 5), suggesting a more balanced repertoire of pathogen recognition receptors capable of responding to diverse microbial challenges including NDV. Similar induction of TLR-mediated pathways following NDV infection or vaccination has been reported previously, emphasizing the importance of innate immune sensing in shaping protective responses against avian paramyxovirus type-1 (Cheng et al., 2014; Neerukonda and Katneni, 2020; Rue et al., 2011).

Interestingly, several crossbred populations (CBD, CSH, BCSML and CR) exhibited comparatively higher expression of one or more TLR genes following ND vaccination. Such enhanced expression may be attributed to heterosis effects arising from the combination of genetically diverse parental lines, which can improve immune competence through increased heterozygosity and favorable allele interactions. Previous studies have demonstrated that crossbreeding can enhance disease resistance and immune responsiveness in poultry by integrating adaptive traits from distinct genetic backgrounds (Jie and Liu, 2011; Lamont et al., 2002). The relatively elevated expression of TLR2A, TLR3, TLR4, and TLR5 observed in certain synthetic broiler lines may reflect differences in genetic background and adaptation to production environments, where effective innate immune responses contribute to disease resistance and flock performance (Broom and Kogut, 2018). The elevated expression of TLRs observed in certain crossbred populations may therefore reflect a synergistic interaction between indigenous disease-resistance traits and the productive genetic background of commercial lines. Genome-wide analyses in chickens have identified several immune-related genes, including innate immune signalling components, within regions subjected to positive selection during breed development and genetic improvement (Berghof et al., 2019; Broom and Kogut, 2019). Since birds were sampled following ND vaccination, the observed differences likely represent breed-specific responsiveness of innate immune pathways rather than merely constitutive TLR expression, suggesting that some crossbred populations may possess enhanced capacity to recognise and respond to viral pathogen-associated molecular patterns.

Among various TLRs studied, TLR3, TLR5, TLR21, TLR2A, and TLR7 formed a closely associated cluster, indicating coordinated regulation of pathogen-recognition pathways involved in innate immune activation. In contrast, TLR4 clustered independently, suggesting a distinct regulatory role primarily associated with inflammatory responses. TLR4 exhibited a distinct loading pattern, contributing mainly to PC2 rather than PC1, indicating that its expression varies independently of the general TLR activation profile and may reflect breed-specific inflammatory regulation rather than antiviral responses. TLR7, TLR5, TLR3, and TLR2A clustered closely, suggesting coordinated transcriptional regulation following vaccination, whereas TLR21 showed a distinct expression pattern despite contributing substantially to overall variance. Broad ellipses for KN, WFRZ, and SFLB indicate greater within-breed variability in TLR expression, likely reflecting higher genetic heterogeneity, while tighter clustering in synthetic populations suggests more uniform innate immune responses.

Among the investigated receptors, TLR7 emerged as one of the most highly expressed genes, highlighting its central role in antiviral immunity. TLR7 is localized in endosomes and recognizes Poly U (Uridine) or GU (Guanosine-Uridine) rich sequences in the viral-derived single stranded (ss) RNA and induces antiviral immunity by up-regulating various pro-inflammatory cytokines and type-I IFNs (Annamalai et al., 2015). Upon activation, TLR7 signals through the MyD88-dependent pathway, leading to activation of NF-κB and IRF7 and subsequent production of type I interferons and inflammatory cytokines. Previous studies have demonstrated significant induction of TLR7 expression in chicken macrophages and PBMCs following NDV infection or stimulation with resiquimod, emphasizing its central role in antiviral immunity (Rue et al., 2011; Annamalai et al., 2015). In addition, stimulation with agonists also conferred protection to infectious bursal disease (Annamalai et al., 2016), Avian influenza (Abdul-Cader et al., 2018) and Infectious bronchitis (Matoo et al., 2018), ND virus (Gupta et al., 2020), emphasizing its importance in recognizing RNA viruses and promoting antiviral states. Our previous study in 3-week-old Kadaknath chickens demonstrated significantly elevated TLR7 expression in peripheral blood mononuclear cells (PBMCs) following resiquimod stimulation compared with broiler and White Leghorn (WLH) chickens (Annamalai et al., 2016). Therefore, the elevated TLR7 expression observed in indigenous and synthetic populations in the present study suggests enhanced viral sensing capacity and may partly explain breed-dependent differences in vaccine responsiveness.

An important finding of the present study was the strong association between TLR3 and TLR7, as demonstrated by both correlation and clustering analyses. TLR3 recognizes double-stranded RNA intermediates produced during viral replication and activates the TRIF-dependent signaling pathway, resulting in IRF3-mediated induction of IFN-β and antiviral genes. The association of TLR3 expression and resistance to Infectious bursal disease (Guo et al., 2012) and Marek’s disease (Hu et al., 2016; Zou et al., 2017) was previously reported. Cheng et al. (2014) demonstrated that stimulation of TLR3 inhibits NDV replication through enhanced production of pro-inflammatory cytokines and type I interferons. The strong co-expression of TLR3 and TLR7 observed in the present investigation suggests synergistic activation of complementary viral recognition mechanisms. Such coordination enables recognition of both genomic single-stranded RNA and replication-associated double-stranded RNA intermediates, thereby amplifying antiviral signaling. Earlier, Raj et al. (2011) demonstrated a higher expression of TLR3 in the spleen of Aseel chicken in comparison to the commercial White Leghorn bird following challenge with very virulent IBDV contributing to the resistance against clinical disease. Contrary to our findings, Ramasamy et al. (2010) reported higher TLR3 expression in heterophils of White Leghorn than indigenous breeds. This difference might be due to change in cells types, vaccination status as well as age of birds used in the experiments. The simultaneous activation of MyD88- and TRIF-dependent pathways may explain the enhanced innate immune responsiveness observed in several indigenous and synthetic populations.

Structural docking analysis provided additional mechanistic support for the association of TLR3 and TLR7 with NDV associated molecular patterns. In the RNA–protein docking analysis, representative RNA fragments derived from the NDV F and HN genes showed more favorable predicted interactions with TLR3 than with TLR7. The F RNA–TLR3 complex exhibited the most favorable docking score (−545.12), followed by HN RNA–TLR3 (−476.88), whereas the corresponding F RNA–TLR7 and HN RNA–TLR7 complexes showed lower scores of −365.45 and −327.00, respectively. All four complexes showed high HDOCK confidence scores (>0.97). Although these computational scores do not represent experimental binding affinities, the consistent preference for TLR3 in both viral RNA analyses is biologically plausible because TLR3 is primarily associated with recognition of double-stranded RNA and replication-associated RNA intermediates, whereas TLR7 preferentially recognizes single-stranded RNA in endosomal compartments. Thus, the docking results provide a structural hypothesis that the RNA structures generated during NDV replication may preferentially engage TLR3, while TLR7 may contribute through recognition of distinct viral RNA species or cellular contexts. This interpretation is consistent with the strong TLR3–TLR7 co-expression observed in the present study and supports the possibility of complementary recognition of viral RNA by these two receptors.

The protein–protein docking analysis further revealed distinct predicted interaction patterns for the major NDV envelope glycoproteins. The NDV F protein exhibited its highest PatchDock score with TLR5 (22,798.0), whereas HN showed its highest score with TLR7 (22,698.0), indicating greater predicted geometric complementarity with these receptors. The F–TLR5 prediction is particularly noteworthy in view of the strong TLR5 expression and its close clustering with TLR3 and TLR7 observed in the present study. However, chicken TLR5 is primarily characterized as a receptor for bacterial flagellin, and therefore the predicted F–TLR5 interaction should not be interpreted as evidence of direct physiological recognition of NDV F protein. Rather, it may represent a potential structural association or receptor cross-talk mechanism that warrants experimental investigation. Similarly, the predicted HN–TLR7 interaction should be interpreted cautiously because TLR7 is principally recognized as an endosomal RNA sensor. Collectively, these findings suggest that the elevated expression and coordinated regulation of TLR3, TLR5 and TLR7 observed following ND vaccination may reflect a broader and interconnected pattern-recognition network rather than isolated receptor responses. Although F–TLR5 and HN–TLR7 exhibited the highest PatchDock scores, the most favorable ACE values were obtained for F–TLR21 and HN–TLR4, respectively. This difference highlights that docking-score rankings based on geometric complementarity and estimated atomic contact energy are not necessarily concordant.

Interestingly, TLR5 clustered closely with TLR3 and TLR7 and exhibited strong positive correlations with these receptors, a pattern that is also noteworthy in light of the predicted F–TLR5 interaction observed in the docking analysis (Fig. 1). Although TLR5 classically recognizes bacterial flagellin in altered fashion (Kannaki et al., 2018; Vaezirad et al., 2018), increasing evidence suggests that TLR signaling pathways are highly interconnected and that activation of one receptor frequently modulates the expression of others through shared signaling molecules including MyD88, MAPKs, NF-κB, and interferon regulatory factors (Keestra et al., 2013). Thus, elevated TLR5 expression may not necessarily indicate direct recognition of NDV but rather represent a generalized state of innate immune preparedness. Similar coordinated upregulation of TLR5 and antiviral receptors has been reported in avian immune cells exposed to microbial stimuli, suggesting extensive cross-talk among pathogen-recognition pathways (Brownlie and Allan, 2011). Such broad activation may enhance resistance against secondary bacterial infections that frequently accompany viral diseases. Moreover, stimulation of PBMCs with TLR5 agonist (flagellin) induced higher expression of pro-inflammatory cytokine IL-1β in Aseel than WLH chickens (Kannaki et al., 2018). Sławińska et al. (2013) reported that TLR5 expression was higher in commercial (Hy-Line) chicken in comparison to indigenous Green-legged Partridge like chickens. The up-regulated ex-pression of TLR2A, TLR4, TLR5 and TLR15 in vagina of mature chicken on exposure to *Salmonella enteritidis* suggested the role of these TLRs in protection against *S. enteritidis* infection (Michailidis et al., 2011).

TLR21 also exhibited strong associations with TLR3 and TLR7, indicating participation in a common receptor network. TLR21 is regarded as the functional avian homolog of mammalian TLR9 and recognizes unmethylated CpG DNA motifs. Activation of TLR21 by CpG-ODN induced pro-inflammatory and Th1 biased immune responses in the neonatal chicken (Brownlie et al., 2009). CpG-ODN also induced naive B-lymphocytes proliferation, survival and enhanced antigen presenting ability to T-lymphocytes through activation of TLR9 signaling cascade (Jiang et al., 2007; Patel et al., 2008). Although NDV is an RNA virus, TLR21 activation may occur indirectly through cytokine-mediated cross-regulation and enhanced antigen presentation. Previous studies have shown that TLR21 stimulation promotes cytokine production and maturation of antigen-presenting cells, thereby facilitating adaptive immune responses (Brownlie and Allan, 2011). The close association of TLR21 with antiviral receptors observed in the present study suggests that multiple pattern-recognition pathways cooperate to amplify vaccine-induced immunity. Such receptor synergy may represent an important mechanism contributing to differences in immunocompetence among chicken populations.

Another noteworthy observation was the strong relationship between TLR2A and antiviral receptors. Although TLR2 is a versatlile receptor role in recognition of wide variety of ligands inlcuding bacterial lipoproteins and endogenous danger-associated molecular patterns, viral infections are known to induce TLR2-mediated inflammatory responses through MyD88-dependent signaling. In chicken, two subtypes of TLR2 (TLR2A and TLR2B) are expressed on cell surface and in-volved in identification of di-acyl and triacyl lipopeptides associated with bacteria, mycobacteria, fungi and yeast cell wall (Keestra et al., 2013). Reduced expression of TLR2A, TLR4 and TLR21 in the peripheral blood leukocytes impaired the host resistance against *S. enteritidis* (Gou et al., 2012). Furthermore, the reduced TLR2 expression in the spleen and cecal tonsils of broiler chicken was associated with the increased susceptibility to *S. enteritidis* (Quinteiro-Filho et al., 2017). Comparatively higher expression of TLR2A in fancy (WFRZ) and indigenous (AR, KN) breeds in the present study indicates that, these breeds might have better host resistance to Salmonella. Activation of TLR2 contributes to production of IL-1β, IL-6, and TNF-α, thereby enhancing recruitment and activation of immune cells. Consequently, elevated TLR2A expression in highly responsive populations may reflect amplification of inflammatory pathways necessary for efficient antigen presentation and initiation of adaptive immunity. The coordinated expression of TLR2A with TLR3, TLR5, and TLR7 further supports the existence of integrated receptor networks underlying vaccine-induced immune responses.

Chicken’s TLR4 recognizes lipopolysaccharides (LPS) found in the cell wall of Gram-negative bacteria (Neerukonda and Katneni, 2020). TLR4 is upregulated in viral infections such as infectious bronchitis and Newcastle disease, indicating their role in development of anti-viral response (Lee et al., 2021; Li et al., 2013). The association of TLR4 with Mx gene in indigenous Senthul chickens conferred resistance to ND and Salmonella (Mamutse et al., 2018). Moreover, the effect of allelic variation of TLR4 on resistance to Salmonella infection in different chicken breeds was previously documented, suggesting TLR4 as a potential candidate for use in selection programme to improve genetic resistance against salmonellosis in chicken (Karnati et al., 2015; Li et al., 2017). In the current study, the expression of TLR4 was found higher in the PBMCs from AR, BCSML and SFLB compared to all other breeds studied. This is supported by the earlier reports of higher constitutive ex-pression of TLR4 in the PBMCs from Aseel breed as compared to other indigenous and broiler breeds (Gupta et al., 2013; Patel et al., 2013). In another study, the expression of TLR4 and TLR5 were found higher in the heterophils from Kadaknath than Aseel (Haunshi et al., 2017; Ramasamy et al., 2011). This may be due to the variation in cell types used in the gene expression study.

This study establishes the first systems-level framework for evaluating innate immune architecture among chicken breeds using integrated TLR indices in response to Genotype-II of the ND vaccine virus in PBMCs. The TLR mediated immunity chicken breeds is characterized by functional specialization rather than hierarchical superiority, with different genetic groups exhibiting distinct investments in pathogen recognition pathways. The strong interrelationships among multiple TLR genes, coupled with breed-specific clustering patterns observed in PCA, indicate that innate immune responsiveness in chickens is governed by coordinated TLR receptor networks whose expression is substantially shaped by genetic background. Of all, TLR4 revealed to be a novel transcript with a distinct expression pattern. Importantly, higher TLR expression should not be interpreted as direct evidence of superior disease resistance, as protective immunity is determined by the coordinated interaction of innate, humoral, and cell-mediated immune mechanisms.

Indigenous breeds, particularly Kadaknath and Aseel, generally exhibited enhanced expression of several TLR transcripts associated with pathogen recognition and antiviral defense, suggesting a potentially greater capacity for rapid innate immune activation. In contrast, exotic layer and broiler breeds displayed distinct receptor-specific expression patterns, reflecting differences that may have arisen through long-term selection for production traits. The observed enhanced expression patterns synthetic and crossbred populations likely result from a combination of heterosis, increased genetic diversity, selective breeding history, and the integration of adaptive traits from indigenous and commercial parental stocks. Such populations may serve as valuable genetic resources for developing disease-resilient poultry lines capable of maintaining productivity under diverse production environments. These results also provide preliminary information for considering indigenous breeds for the development of location and disease specific low input technology birds in a crossbreeding programme to achieve prosperity in rural poultry production.

The composite immune indices proposed herein, including the VII, VRI BRI, IDS, Shannon TLR Diversity Index (STDI), and IBI, should be regarded as hypothesis-generating biomarkers rather than definitive correlates of protection. PC1 weights reflect covariance in this dataset, not receptor importance. VRI and BRI are positively correlated (p = 0.73-0.81), so they largely track overall TLR expression and should not be read as independent antiviral vs. antibacterial axes. We warrant further studies involving independent populations, longitudinal studies, virulent NDV challenge models, TLR-based immune stimulation, and integrated multi-omics analyses etc. Nevertheless, these indices provide a quantitative framework for characterizing breed-specific innate immune architecture and represent promising tools for precision breeding, vaccine evaluation, and immunogenetic research in poultry. Among the RNA–TLR complexes evaluated, the representative F- and HN-gene-derived RNA fragments showed more favorable predicted docking with TLR3 than with TLR7, identifying TLR3 as a candidate receptor for further experimental investigation of NDV-associated RNA sensing.

The TLR expression profiles observed in the present study reflect the transcriptional state of the birds at 48 h following LaSota vaccination. However, because breed-matched unvaccinated controls were not included, the observed differences cannot be unequivocally attributed to vaccine-induced upregulation. In particular, the relatively higher TLR expression observed in Aseel Red and Kadaknath may reflect inherent breed-associated differences in basal immune gene expression, differential responsiveness to vaccination, or both. Thus, the present findings should be interpreted as breed-associated TLR expression patterns following a standardized ND vaccination challenge, rather than as direct evidence of differences in vaccine-induced fold induction or immune responsiveness. Future studies incorporating breed-matched unvaccinated controls, baseline sampling, and multiple post-vaccination time points would be required to distinguish constitutive expression differences from vaccine-induced transcriptional responses.

## Author contributions

**Nikunj Kumar Patel** conducted laboratory experiments, generated research data, performed statistical analyses, interpreted the results, and contributed to the preparation of the original manuscript. **Gautham Kolluri** conceived the research idea, designed the study, developed and coordinated the experimental methodology, contributed to data acquisition, performed statistical analyses, interpreted the findings, and prepared the original manuscript. **Abhinaya Kaliappan** conducted laboratory experiments, including gene-expression analysis, and contributed to data acquisition and interpretation. **Shiva Kumar** contributed to sample collection, performed formal data analysis, organized data and interpreted research findings. **Aditi Vats** contributed to data acquisition, data curation, computational and software-based analyses, and interpretation of the findings. **Saravanan Ramakrishna** contributed to the conceptualization and methodological design of the study, interpretation of the findings, and critical revision of the manuscript for important intellectual content. **Jagbir Singh Tyagi** secured research funding, contributed to the interpretation of the research findings and critically reviewed and edited the revised manuscript for important intellectual content. All authors made substantial contributions to the research through the activities described above. All authors critically reviewed the original and revised manuscripts for important intellectual content, explicitly approved the final version submitted for publication, and agreed to be personally accountable for all aspects of the work, including ensuring that any questions concerning the accuracy or integrity of the research are appropriately investigated and resolved.

## Data availability

The raw datasets generated and/or analysed during the current study are not publicly available due to prior permission issues but are available from the corresponding author on reasonable request.

## Funding

This research was funded by Department of Biotechnology, Government of India, grant number BT/PR32656/AAQ/1/763/2019. The financial support of Indian Council of Agricultural Research is also duly acknowledged.

## Declaration of generative AI and AI-assisted technologies in the manuscript preparation process

During the preparation of this work, the author(s) used Open AI (Chat GPT May version 2026) for english language editing, paraphrasing, and enhancement of visibility and designing of data figures as an input. The author(s) reviewed and edited the output as needed and take full responsibility for the content of the published article.

## Disclosures

The authors have no known competing financial interests or personal relationships that could have appeared to influence the work reported in this paper.

## Footnotes

Supplementary material associated with this article can be found, in the online version, at doi:10.1016/j.psj.2026.107908.

## References

[bib0001] Abdul-Cader M.S., Senapathi U.D.S., Nagy E., Sharif S., Abdul-Careem M.F. (2018). Antiviral response elicited against avian influenza virus infection following activation of toll-like receptor (TLR) 7 signaling pathway is attributable to interleukin (IL)-1β production. BMC Res. Notes..

[bib0002] Akira S., Uematsu S., Takeuchi O. (2006). Pathogen recognition and innate immunity. Cell.

[bib0003] Alexander D.J. (2001). Newcastle disease. Br. Poult. Sci..

[bib0004] Annamalai A., Ramakrishnan S., Sachan S., Kumar B.A., Sharma B.K., Kumar V., Palanivelu M., Varghese B.P., Kumar A., Saravanan B.C., Krishnaswamy N. (2016). Prophylactic potential of resiquimod against very virulent infectious bursal disease virus (vvIBDV) challenge in the chicken. Vet. Microbiol..

[bib0005] Annamalai A., Ramakrishnan S., Sachan S., Sharma B.K., Kumar B.A., Kumar V., Badasara S.K., Kumar A., Saravanan B.C., Krishnaswamy N. (2015). Administration of TLR7 agonist, resiquimod, in different types of chicken induces a mixed Th1 and Th2 response in the peripheral blood mononuclear cells. Res. Vet. Sci..

[bib0006] Bashir K., Kappala D., Singh Y., Dar J.A., Mariappan A.K., Kumar A., Krishnaswamy N., Dey S., Chellappa M.M., Goswami T.K., Gupta V.K. (2019). Combination of TLR2 and TLR3 agonists derepress infectious bursal disease virus vaccine-induced immunosuppression in the chicken. Sci. Rep..

[bib0007] Berghof T.V.L., Matthijs M.G.R., Arts J.A.J., Bovenhuis H., Dwars R.M., Van der Poel J.J., Visker M.H.P.W., Parmentier H.K. (2019). Selective breeding for high natural antibody level increases resistance to avian pathogenic *Escherichia coli* (APEC) in chickens. Dev. Comp. Immunol..

[bib0008] Broom L.J., Kogut M.H. (2018). Gut immunity: its development and reasons and opportunities for modulation in monogastric production animals. Anim. Health Res. Rev..

[bib0009] Broom L.J., Kogut M.H. (2019). Deciphering desirable immune responses from disease models with resistant and susceptible chickens. Poult. Sci..

[bib0010] Brownlie R., Allan B. (2011). Avian toll -like receptors. Cell Tissue Res..

[bib0011] Brownlie R., Zhu J., Allan B., Mutwiri G.K., Babiuk L.A., Potter A., Griebel P. (2009). Chicken TLR21 acts as a functional homologue to mammalian TLR9 in the recognition of CpG oligodeoxynucleotides. Mol. Immunol..

[bib0012] Chen S., Cheng A., Wang M. (2013). Innate sensing of viruses by pattern recognition receptors in birds. Vet. Res..

[bib0013] Cheng H.W., Muir W.M. (2005). The effects of genetic selection for survivability and productivity on chicken physiological homeostasis. World's Poult. Sci. J..

[bib0014] Cheng J., Sun Y., Zhang X., Zhang F., Zhang S., Yu S., Qiu X., Tan L., Song C., Gao S., Wu Y. (2014). Toll -like receptor 3 inhibits Newcastle disease virus replication through activation of pro-inflammatory cytokines and the type-1 interferon pathway. Arch. Virol..

[bib0015] Emam M., Mehrabani-Yeganeh H., Barjesteh N., Nikbakht G., Thompson-Crispi K., Charkhkar S., Mallard B. (2014). The influence of genetic background versus commercial breeding programs on chicken immunocompetence. Poult. Sci..

[bib0016] Girija S., Kolluri G., Sharma S., Tyagi J.S., Ansari M.M., Singh T., Kaliappan A., Tiwari A.K. (2023). Assessing cytokine responses in indigenous and exotic chicken breeds following velogenic Newcastle disease virus challenge. Indian J. Poult. Sci..

[bib0017] Girija S., Tyagi J.S., Mariappan A.S., Rahman F.A.T., Sharma S., Singh T., Srivastava V., Kolluri G. (2023). Studies on production and biochemical profile in indigenous and exotic chicken challenged with velogenic Newcastle disease virus (vNDV). Indian J. Poult. Sci..

[bib0018] Gou Z., Liu R., Zhao G., Zheng M., Li P., Wang H., Zhu Y., Chen J., Wen J. (2012). Epigenetic modification of TLRs in leukocytes is associated with increased susceptibility to *Salmonella enteritidis* in chickens. PLoS One.

[bib0019] Guo X., Rosa A.J.M., Chen D.G., Wang X. (2012). Differential expression of the toll -like receptor pathway and related genes of chicken bursa after experimental infection with infectious bursa disease virus. Arch. Virol..

[bib0020] Gupta A., Deka P., Kumar S. (2020). Resiquimod inhibits Newcastle disease virus replication by modulating host cytokines, an understanding towards its possible therapeutics. Cytokine.

[bib0021] Gupta S.K., Deb R., Chellappa M.M., Dey S. (2013). Recombinant flagellin and its cross-talk with lipopolysaccharide–effect on pooled chicken peripheral blood mononuclear cells. Res. Vet. Sci..

[bib0022] Gupta S.K., Deb R., Dey S., Chellappa M.M. (2015). Toll -like receptor ligands and their combinations as adjuvants-current research and its relevance in chickens. Worlds Poult. Sci. J..

[bib0023] Gupta V., Bhattacharyya A., Hwang Y.J., Choi Y.H. (2025). *In ovo* sericin suppresses hepatic DNA demethylation in broilers at hatch. Poult. Sci..

[bib0024] Gupta V., Ncho C.M., Goel A., Jeong C.M., Choi Y.H. (2022). Effects of *in ovo* injection of α-ketoglutaric acid on hatchability, growth, plasma metabolites, and antioxidant status of broilers. Antioxidants.

[bib0025] Haunshi S., Burramsetty A.K., Kannaki T.R., Ravindra K.R., Chatterjee R.N. (2017). Pattern recognition receptor genes expression profiling in indigenous chickens of India and White Leghorn. Poult. Sci..

[bib0026] Higuchi M., Matsuo A., Shingai M., Shida K., Ishii A., Funami K., Suzuki Y., Oshiumi H., Matsumoto M., Seya T. (2008). Combinational recognition of bacterial lipoproteins and peptidoglycan by chicken toll -like receptor 2 subfamily. Dev. Comp. Immunol..

[bib0027] Hu X., Zou H., Qin A., Qian K., Shao H., Ye J. (2016). Activation of toll -like receptor 3 inhibits Marek’s disease virus infection in chicken embryo fibroblast cells. Arch. Virol..

[bib0028] Jiang W., Lederman M.M., Harding C.V., Rodriguez B., Mohner R.J., Sieg S.F. (2007). TLR9 stimulation drives naive B cells to proliferate and to attain enhanced antigen presenting function. Eur. J. Immunol..

[bib0029] Jie H., Liu Y.P. (2011). Breeding for disease resistance in poultry, opportunities with challenges. Worlds Poult. Sci. J..

[bib0087] Kaiser P. (2010). Advances in avian immunology—prospects for disease control: a review. Avian Pathol..

[bib0030] Kannaki T.R., Reddy M.R., Ravindra K.S., Chatterjee R.N. (2018). Molecular and functional characterization of toll -like receptor 5 (TLR5) in Aseel and White Leghorn chicken. Indian J. Anim. Res..

[bib0031] Kannaki T.R., Reddy M.R., Verma P.C., Shanmugam M. (2015). Differential toll -like receptor (TLR) mRNA expression patterns during chicken embryological development. Anim. Biotechnol..

[bib0032] Kannaki T.R., Shanmugam M., Verma P.C. (2011). Toll -like receptors and their role in animal reproduction. Anim. Reprod. Sci..

[bib0033] Karnati H.K., Pasupuleti S.R., Kandi R., Undi R.B., Sahu I., Kannaki T.R., Subbiah M., Gutti R.K. (2015). TLR-4 signalling pathway: myD88 independent pathway up-regulation in chicken breeds upon LPS treatment. Vet. Res. Commun..

[bib0034] Kawai T., Akira S. (2010). The role of pattern-recognition receptors in innate immunity, update on toll -like receptors. Nat. Immunol..

[bib0035] Kawai T., Ikegawa M., Ori D., Akira S. (2024). Decoding toll -like receptors, recent insights and perspectives in innate immunity. Immunity.

[bib0037] Keestra A.M., de Zoete M.R., Bouwman L.I., Vaezirad M.M., van Putten J.P.M. (2013). Unique features of chicken toll -like receptors. Dev. Comp. Immunol..

[bib0038] Keestra A.M., de Zoete M.R., Bouwman L.I., van Putten J.P.M. (2013). Chicken TLR21 is an innate CpG DNA receptor distinct from mammalian TLR9. Nat. Commun..

[bib0039] Keestra A.M., de Zoete M.R., van Aubel R.A.M.H., van Putten J.P.M. (2008). Functional characterization of chicken TLR5 reveals species-specific recognition of flagellin. Mol. Immunol..

[bib0040] Kogut M.H., Swaggerty C., He H., Pevzner I., Kaiser P. (2006). Toll -like receptor agonists stimulate differential functional activation and cytokine and chemokine gene expression in heterophils isolated from chickens with differential innate responses. Microbes Infect.

[bib0041] Kolluri G., Katam S.K., Manivasagam V., Tiwari A.K. (2024). Bolstering poultry health, the urgency of updating Newcastle disease virus (NDV) vaccines in India. Preprints.

[bib0042] Kolluri G., Ramamurthy N., Churchil R.R., Dhinakar Raj G., Kannaki T.R. (2014). Influence of age, sex and rearing systems on toll -like receptor 7 (TLR7) expression pattern in gut, lung and lymphoid tissues of indigenous ducks. Br. Poult. Sci..

[bib0088] Lamont S.J., Kaiser M.G., Liu W. (2002). Candidate genes for resistance to Salmonella enteritidis colonization in chickens as detected in a novel genetic cross. Vet. Immunol. Immunopathol..

[bib0044] Lee R., Jung J.S., Yeo J.I., Kwon H.M., Park J. (2021). Transcriptome analysis of primary chicken cells infected with infectious bronchitis virus strain K047-12 isolated in Korea. Arch. Virol..

[bib0045] Li H.F., Zhu W.Q., Chen K.W., Wu X., Tang Q.P., Gao Y.S. (2013). Genetic differences in TLR4 gene polymorphisms and expression involved in Salmonella natural and artificial infection respectively in Chinese native chicken breeds. Mol. Biol. Rep..

[bib0046] Li P., Wang Y., Li H., Zheng M., Wen J., Zhao G. (2017). Allelic variation in TLR4 is linked to resistance to *Salmonella Enteritidis* infection in chickens. Poult. Sci..

[bib0047] Livak K.J., Schmittgen T.D. (2001). Analysis of relative gene expression data using real-time quantitative PCR and the 2−ΔΔCT method. Methods.

[bib0048] Lochmiller R.L., Deerenberg C. (2000). Trade-offs in evolutionary immunology, just what is the cost of immunity?. Oikos.

[bib0049] Looi F.Y., Baker M.L., Townson T., Richard M., Novak B., Doran T.J., Short K.R. (2018). Creating disease resistant chickens: a viable solution to avian influenza?. Viruses.

[bib0050] MacKinnon K.M., He H., Swaggerty C.L., Genovese K.J., Kogut M.H. (2009). Expression profile of toll -like receptors within the gastrointestinal tract of 2-day-old *Salmonella Enteritidis*-infected broiler chickens. Vet. Microbiol..

[bib0051] Mamutse J., Gunawan A., Sumantri C., Murtini S., Sartika T. (2018). Association of the toll -like receptor 4 (TLR4) and myxovirus (Mx) genes with resistance to *Salmonella* and Newcastle disease in selected Sentul chickens. Int. J. Poult. Sci..

[bib0052] Matoo J.J., Bashir K., Kumar A., Krishnaswamy N., Dey S., Chellappa M.M., Ramakrishnan S. (2018). Resiquimod enhances mucosal and systemic immunity against avian infectious bronchitis virus vaccine in the chicken. Microb. Pathog..

[bib0053] Michailidis G., Theodoridis A., Avdi M. (2011). Effects of sexual maturation and *Salmonella* infection on the expression of toll -like receptors in the chicken vagina. Anim. Reprod. Sci..

[bib0054] Naldurtiker A., Batchu P., Kouakou B., Terrill T.H., McCommon G.W., Kannan G. (2023). Differential gene expression analysis using RNA-seq in the blood of goats exposed to transportation stress. Sci. Rep..

[bib0055] Nang N.T., Lee J.S., Song B.M., Kang Y.M., Kim H.S., Seo S.H. (2011). Induction of inflammatory cytokines and toll -like receptors in chickens infected with avian H9N2 influenza virus. Vet. Res..

[bib0056] Nawab A., An L., Wu J., Li G., Liu W., Zhao Y., Wu Q., Xiao M. (2019). Chicken toll -like receptors and their significance in immune response and disease resistance. Int. Rev. Immunol..

[bib0057] Neerukonda S.N., Katneni U. (2020). Avian pattern recognition receptor sensing and signaling. Vet. Sci..

[bib0058] Ortiz-Burgos S. (2015). Shannon-weaver diversity index. Encycl. Earth Sci. Ser..

[bib0059] Patel B.A., Gomis S., Dar A., Willson P.J., Babiuk L.A., Potter A., Mutwiri G., Tikoo S.K. (2008). Oligodeoxynucleotides containing CpG motifs (CpG-ODN) predominantly induce Th1-type immune response in neonatal chicks. Dev. Comp. Immunol..

[bib0060] Patel M.V., Kumar P., Das H., Dadawala A., Chauhan H.C., Chandel B.S., Shah N.M., Kher H.N. (2013). Evaluation of the constitutive expression levels of ch-TLR3, ch-TLR4, ch-TLR15 and ch-TLR21 genes in the peripheral blood mononuclear cells of native Indian poultry breeds. Aseel and Kadaknath. Vet. World..

[bib0061] Pfaffl M.W., Tichopad A., Prgomet C., Neuvians T.P. (2004). Determination of stable housekeeping genes, differentially regulated target genes and sample integrity, BestKeeper–Excel-based tool using pair-wise correlations. Biotechnol. Lett..

[bib0062] Prabhu S.N., Singh A.P., Varghese B.P., Dhama K., Singh S.D., Singh R. (2020). A comparative study of pathology and host immune response induced by very virulent infectious bursal disease virus in experimentally infected chickens of Aseel and White Leghorn breeds. Vaccines..

[bib0063] Quan R., Zhu S., Wei L., Wang J., Yan X., Li Z., Liu J. (2017). Transcriptional profiles in bursal B-lymphoid DT40 cells infected with very virulent infectious bursal disease virus. Virol. J..

[bib0064] Quinteiro-Filho W.M., Calefi A.S., Cruz D.S.G., Aloia T.P.A., Zager A., Astolfi-Ferreira C.S., Ferreira J.P., Sharif S., Palermo-Neto J. (2017). Heat stress decreases expression of the cytokines, avian β-defensins 4 and 6 and toll -like receptor 2 in broiler chickens infected with *Salmonella Enteritidis*. Vet. Immunol. Immunopathol..

[bib0065] R Core Team (2020).

[bib0066] Raj G.D., Rajanathan T.C., Kumanan K., Elankumaran S. (2011). Changes in the cytokine and toll -like receptor gene expression following infection of indigenous and commercial chickens with infectious bursal disease virus. Indian J. Virol..

[bib0067] Rajkumar U., Haunshi S., Paswan C., Raju M.V.L.N., Rao S.R., Chatterjee R.N. (2017). Characterization of indigenous Aseel chicken breed for morphological, growth, production, and meat composition traits from India. Poult. Sci..

[bib0068] Ramasamy K.T., Reddy M.R., Murugesan S. (2011). Toll-like receptor mRNA expression, iNOS gene polymorphism and serum nitric oxide levels in indigenous chickens. Vet. Res. Commun..

[bib0069] Ramasamy K.T., Reddy M.R., Raveendranathan D.N., Murugesan S., Chatterjee R.N., Ullengala R., Haunshi S. (2010). Differential expression of toll -like receptor mRNA in White Leghorn and indigenous chicken of India. Vet. Res. Commun..

[bib0072] Rue C.A., Susta L., Cornax I., Brown C.C., Kapczynski D.R., Suarez D.L., King D.J., Miller P.J., Afonso C.L. (2011). Virulent Newcastle disease virus elicits a strong innate immune response in chickens. J. Gen. Virol..

[bib0073] Schmid-Hempel P. (2003). Variation in immune defence as a question of evolutionary ecology. Proc. R. Soc. B Biol. Sci..

[bib0074] Sheldon B.C., Verhulst S. (1996). Ecological immunology, costly parasite defences and trade-offs in evolutionary ecology. Trends Ecol. Evol..

[bib0075] Silver N., Best S., Jiang J., Thein S.L. (2006). Selection of housekeeping genes for gene expression studies in human reticulocytes using real-time PCR. BMC Mol. Biol..

[bib0076] Sławińska A., D’Andrea M., Pilla F., Bednarczyk M., Siwek M. (2013). Expression profiles of toll -like receptors 1, 2 and 5 in selected organs of commercial and indigenous chickens. J. Appl. Genet..

[bib0077] Spellerberg I.F., Fedor P.J. (2003). A tribute to Claude Shannon (1916–2001) and a plea for more rigorous use of species richness, species diversity and the ‘Shannon–Wiener’Index. Global Ecol. biogeogr..

[bib0078] St Paul M., Paolucci S., Barjesteh N., Wood R.D., Sharif S. (2013). Chicken erythrocytes respond to toll -like receptor ligands by up-regulating cytokine transcripts. Res. Vet. Sci..

[bib0079] Świderská Z., Šmídová A., Buchtová L., Bryjová A., Fabiánová A., Munclinger P., Vinkler M. (2018). Avian toll -like receptor allelic diversity far exceeds human polymorphism: an insight from domestic chicken breeds. Sci. Rep..

[bib0080] Vaezirad M.M., Koene M.G.J., Wagenaar J.A., van Putten J.P.M. (2018). Chicken immune response following *in ovo* delivery of bacterial flagellin. Vaccine.

[bib0081] Vanamamalai V.K., Garg P., Kolluri G., Gandham R.V., Jali I., Sharma S. (2021). Transcriptomic analysis to infer key molecular players involved during host response to NDV challenge in Gallus gallus (Leghorn & Fayoumi). Sci. Rep..

[bib0082] Vandesompele J., De Preter K., Pattyn F., Poppe B., Van Roy N., De Paepe A., Speleman F. (2002). Accurate normalization of real-time quantitative RT-PCR data by geometric averaging of multiple internal control genes. Genome Biol..

[bib0084] Yadav S.P., Kannaki T.R., Mahapatra R.K., Paswan C., Bhattacharya T.K., Sarkar S.K., Chatterjee R.N. (2018). In vivo cell-mediated immune, hemagglutination inhibition response, hematological and biochemical values in native vs. exotic chicken breeds. Poult. Sci..

[bib0085] Yang F., Lei X., Rodriguez-Palacios A., Tang C., Yue H. (2013). Selection of reference genes for quantitative real-time PCR analysis in chicken embryo fibroblasts infected with avian leukosis virus subgroup J. BMC Res. Notes..

[bib0086] Zou H., Su R., Ruan J., Shao H., Qian K., Ye J., Qin A. (2017). Toll-like receptor 3 pathway restricts Marek’s disease virus infection. Oncotarget.

