# Supplementary material for: Comparative Toll-like receptor profiling reveals breed-specific innate immune architecture following Newcastle disease vaccination
Source: Poult Sci. 2026 Oct 5;105(12):107908. doi: 10.1016/j.psj.2026.107908 (PMC13677765; doi:10.1016/j.psj.2026.107908)
Supplement: Supplementary file 1 [file mmc1.docx]

**Supplemental** **Table 1.** Breed-level variation in the VRI and BRI under alternative weighting schemes

|  | **VRI** | | | | **BRI** | |
| --- | --- | --- | --- | --- | --- | --- |
|  | **Equal raw weights** | **Equal z weights** | **Pc1 loading weights** | **RNA sensing only weights** | **Equal raw weights** | **Pc1 loading weights** |
| WLH | 0.000 | -0.354 | -0.356 | -0.321 | 0.000 | -0.325 |
| RIR | -0.050 | -0.405 | -0.384 | -0.326 | -0.421 | -0.811 |
| CR | 0.521 | 0.308 | 0.222 | -0.050 | 0.098 | -0.199 |
| AR | 0.687 | 0.534 | 0.637 | 1.007 | 1.252 | 1.247 |
| AB | -0.144 | -0.527 | -0.474 | -0.280 | -0.139 | -0.393 |
| KN | 1.171 | 1.160 | 1.148 | 1.040 | 0.279 | 0.299 |
| NCB | -0.238 | -0.659 | -0.663 | -0.630 | -0.471 | -0.892 |
| CSH | 0.653 | 0.492 | 0.533 | 0.662 | 0.648 | 0.588 |
| CBD | 0.412 | 0.166 | 0.192 | 0.362 | 0.518 | 0.239 |
| BCSML | 0.085 | -0.239 | -0.279 | -0.419 | 0.571 | 0.235 |
| BCSFL | 0.859 | 0.752 | 0.773 | 0.841 | 0.682 | 0.432 |
| SFLB | -1.016 | -1.629 | -1.658 | -1.828 | -0.467 | -1.138 |
| FRZ | 0.446 | 0.234 | 0.260 | 0.308 | 0.469 | 0.238 |
| WFRZ | 0.611 | 0.433 | 0.344 | 0.011 | 0.523 | 0.651 |
| SLK | 0.061 | -0.268 | -0.291 | -0.377 | 0.184 | -0.172 |

For VRI, equal raw weights, equal Z-score weights, principal component 1 (PC1) loading weights, and RNA-sensing pathway-only weights were evaluated. For BRI, equal raw weights and PC1 loading weights were evaluated. VRI: Viral-sensing receptor index; BRI: Bacterial sensing receptor index.

**Supplemental** **Table 2.** Comparison of breed rankings based on VRI under alternative weighting schemes

|  | **Equal raw weights** | **Equal Z weights** | **Pc1 loading weights** | **RNA sensing only weights** |
| --- | --- | --- | --- | --- |
| WLH | 11 | 11 | 11 | 10 |
| RIR | 12 | 12 | 12 | 11 |
| CR | 6 | 6 | 7 | 8 |
| AR | 3 | 3 | 3 | 2 |
| AB | 13 | 13 | 13 | 9 |
| KN | 1 | 1 | 1 | 1 |
| NCB | 14 | 14 | 14 | 14 |
| CSH | 4 | 4 | 4 | 4 |
| CBD | 8 | 8 | 8 | 5 |
| BCSML | 9 | 9 | 9 | 13 |
| BCSFL | 2 | 2 | 2 | 3 |
| SFLB | 15 | 15 | 15 | 15 |
| FRZ | 7 | 7 | 6 | 6 |
| WFRZ | 5 | 5 | 5 | 7 |
| SLK | 10 | 10 | 10 | 12 |

Values represent the mean rank of each breed based on the VRI calculated using the indicated weighting scheme (n = 6 per breed). Lower rank values indicate a higher relative VRI ranking.

Abbreviations: WLH, White Leghorn; RIR, Rhode Island Red; CR, Cornish; AR, Australorp; AB, Aseel Black; KN, Kadaknath; NCB, Naked Neck Black; CSH, CARI Shyama; CBD, Cobb; BCSML, Black Chicken Small; BCSFL, Black Chicken Small Featherless; SFLB, Silky Featherless Black; FRZ, Frizzle; WFRZ, White Frizzle; SLK, Silkie.

**Supplemental** **Table 3: Bootstrap estimates and 95% confidence intervals of principal component 1 (PC1) loadings for Toll-like receptor genes**

|  | Loading | Lower95 | Median | Upper95 |
| --- | --- | --- | --- | --- |
| TLR2A | 0.442 | 0.394 | 0.44 | 0.471 |
| TLR3 | 0.451 | 0.403 | 0.451 | 0.487 |
| TLR4 | 0.208 | 0.02 | 0.204 | 0.331 |
| TLR5 | 0.451 | 0.403 | 0.451 | 0.513 |
| TLR7 | 0.494 | 0.466 | 0.493 | 0.524 |
| TLR21 | 0.332 | 0.174 | 0.335 | 0.416 |

C1: Principal component 1; TLR: Toll-like receptor

**Supplemental** **Table 4:** Predicted secondary-structure composition of NDV proteins and chicken Toll-like receptors

|  | **F** | **HN** | **TLR2A** | **TLR3** | **TLR4** | **TLR5** | **TLR7** |
| --- | --- | --- | --- | --- | --- | --- | --- |
| Alpha helix (Hh) | 245 (44.30%) | 108 (18.72%) | 394 (49.68%) | 452 (53.81%) | 404 (47.92%) | 416 (48.32%) | 521 (49.20%) |
| 3_10_ helix (Gg) | 0  (0.00%) | 0  (0.00%) | 0  (0.00%) | 0 (0.00%) | 0 (0.00%) | 0 (0.00%) | 0  (0.00%) |
| Pi helix (Ii) | 0  (0.00%) | 0  (0.00%) | 0  (0.00%) | 0 (0.00%) | 0 (0.00%) | 0 (0.00%) | 0  (0.00%) |
| Beta bridge (Bb) | 0  (0.00%) | 0  (0.00%) | 0  (0.00%) | 0 (0.00%) | 0 (0.00%) | 0 (0.00%) | 0  (0.00%) |
| Extended strand (Ee) | 109 (19.71%) | 140 (24.26%) | 94 (11.85%) | 79 (9.40%) | 103 (12.22%) | 100 (11.61%) | 92 (8.69%) |
| Beta turn (Tt) | 0  (0.00%) | 0  (0.00%) | 0  (0.00%) | 0 (0.00%) | 0 (0.00%) | 0 (0.00%) | 0  (0.00%) |
| Bend region (Ss) | 0  (0.00%) | 0  (0.00%) | 0  (0.00%) | 0 (0.00%) | 0 (0.00%) | 0 (0.00%) | 0  (0.00%) |
| Random coil (Cc) | 199 (35.99%) | 329 (57.02%) | 305 (38.46%) | 309 (36.79%) | 336 (39.86%) | 345 (40.07%) | 446 (42.12%) |
| Ambiguous states (?) | 0  (0.00%) | 0  (0.00%) | 0 (0.00%) | 0 (0.00%) | 0 (0.00%) | 0 (0.00%) | 0  (0.00%) |
| Other states | 0  (0.00%) | 0  (0.00%) | 0 (0.00%) | 0 (0.00%) | 0 (0.00%) | 0 (0.00%) | 0  (0.00%) |

Secondary structures were predicted using SOPMA with a window width of 17, similarity threshold of 8, and three-state prediction setting. Percentages represent the proportion of amino acid residues assigned to each predicted structural category. No ambiguous or other structural states were predicted.
