# Supplementary material for: Comparative Toll-like receptor profiling reveals breed-specific innate immune architecture following Newcastle disease vaccination
Source: Poult Sci. 2026 Oct 5;105(12):107908. doi: 10.1016/j.psj.2026.107908 (PMC13677765; doi:10.1016/j.psj.2026.107908)
Supplement: Supplementary file 2 [file mmc2.docx]

Supplemental Fig.1: Sperman’s correlation of equal-weighted RNA sensing only TLRs (TLR3, TLR7) vs PC1 loading’s weighted (TLR3, TLR7, TLR21) breed mean VRI. VRI: Viral-sensing Receptor Index; TLR: Toll-like receptor

Supplemental Fig.2: Breed wise rankings of Viral-sensing Receptor Index (VRI) calculated using PC1 loadings from the PCA as weights on standardized expression values (TLR3 = 0.353, TLR7 = 0.387, TLR21 = 0.260); equal raw weights and RNA sensing only TLRs.
